# EADSG Guidelines: Insulin Therapy in Diabetes

**DOI:** 10.1007/s13300-018-0384-6

**Published:** 2018-03-05

**Authors:** Bahendeka Silver, Kaushik Ramaiya, Swai Babu Andrew, Otieno Fredrick, Sarita Bajaj, Sanjay Kalra, Bavuma M. Charlotte, Karigire Claudine, Anthony Makhoba

**Affiliations:** 1 MKPGMS-Uganda Martyrs University | St. Francis Hospital, Nsambya, Kampala, Uganda; 2 Shree Hindu Mandal Hospital, Chusi Street, Dar es Salaam, Tanzania; 3 Muhimbili University College of Health Sciences, United Nations Road, Dar es Salaam, Tanzania; 4 Department of Clinical Medicine and Therapeutics School of Medicine, College of Health Science, University of Nairobi, Nairobi, Kenya; 5 Department of Medicine, MLN Medical College, George Town, Allahabad, India; 6 Bharti Research Institute of Diabetes and Endocrinology, Sector 12, PO Box 132001, Karnal, Haryana India; 7 University of Rwanda, College of Medicine and Health Science, Kigali University Teaching Hospital, Kigali, Rwanda; 8 Department of Internal Medicine, Rwanda Military Hospital, Kigali, Rwanda

**Keywords:** Diabetes mellitus, East Africa, Guidelines, Hyperglycemia, Hypoglycemia, Insulin therapy, Type 1 diabetes mellitus (T1DM), Type 2 diabetes mellitus (T2DM)

## Abstract

- A diagnosis of diabetes or hyperglycemia should be confirmed prior to ordering, dispensing, or administering insulin **(A)**.
- Insulin is the primary treatment in all patients with type 1 diabetes mellitus (T1DM) **(A)**.
- Typically, patients with T1DM will require initiation with multiple daily injections at the time of diagnosis. This is usually short-acting insulin or rapid-acting insulin analogue given 0 to 15 min before meals together with one or more daily separate injections of intermediate or long-acting insulin. Two or three premixed insulin injections per day may be used **(A)**.
- The target glycated hemoglobin A1c (HbA1c) for all children with T1DM, including preschool children, is recommended to be < 7.5% (< 58 mmol/mol). The target is chosen aiming at minimizing hyperglycemia, severe hypoglycemia, hypoglycemic unawareness, and reducing the likelihood of development of long-term complications **(B)**.
- For patients prone to glycemic variability, glycemic control is best evaluated by a combination of results with self-monitoring of blood glucose (SMBG) **(B)**.
- Indications for exogenous insulin therapy in patients with type 2 diabetes mellitus (T2DM) include acute illness or surgery, pregnancy, glucose toxicity, contraindications to or failure to achieve goals with oral antidiabetic medications, and a need for flexible therapy **(B)**.
- In T2DM patients, with regards to achieving glycemic goals, insulin is considered alone or in combination with oral agents when HbA1c is ≥ 7.5% (≥ 58 mmol/mol); and is essential for treatment in those with HbA1c ≥ 10% (≥ 86 mmol/mol), when diet, physical activity, and other antihyperglycemic agents have been optimally used **(B)**.
- The preferred method of insulin initiation in T2DM is to begin by adding a long-acting (basal) insulin or once-daily premixed/co-formulation insulin or twice-daily premixed insulin, alone or in combination with glucagon-like peptide-1 receptor agonist (GLP-1 RA) or in combination with other oral antidiabetic drugs (OADs) **(B)**.
- If the desired glucose targets are not met, rapid-acting or short-acting (bolus or prandial) insulin can be added at mealtime to control the expected postprandial raise in glucose. An insulin regimen should be adopted and individualized but should, to the extent possible, closely resemble a natural physiologic state and avoid, to the extent possible, wide fluctuating glucose levels **(C)**.
- Blood glucose monitoring is an integral part of effective insulin therapy and should not be omitted in the patient’s care plan. Fasting plasma glucose (FPG) values should be used to titrate basal insulin, whereas both FPG and postprandial glucose (PPG) values should be used to titrate mealtime insulin **(B)**.
- Metformin combined with insulin is associated with decreased weight gain, lower insulin dose, and less hypoglycemia when compared with insulin alone **(C)**.
- Oral medications should not be abruptly discontinued when starting insulin therapy because of the risk of rebound hyperglycemia **(D)**.
- Analogue insulin is as effective as human insulin but is associated with less postprandial hyperglycemia and delayed hypoglycemia **(B)**.
- The shortest needles (currently the 4-mm pen and 6-mm syringe needles) are safe, effective, and less painful and should be the first-line choice in all patient categories; intramuscular (IM) injections should be avoided, especially with long-acting insulins, because severe hypoglycemia may result; lipohypertrophy is a frequent complication of therapy that distorts insulin absorption, and therefore, injections and infusions should not be given into these lesions and correct site rotation will help prevent them **(A)**.
- Many patients in East Africa reuse syringes for various reasons, including financial. This is not recommended by the manufacturer and there is an association between needle reuse and lipohypertrophy. However, patients who reuse needles should not be subjected to alarming claims of excessive morbidity from this practice **(A)**.
- Health care authorities and planners should be alerted to the risks associated with syringe or pen needles 6 mm or longer in children **(A)**.

## Introduction

Based on the most recent International Diabetes Federation (IDF) report, the number of people with diabetes will increase from 425 million in 2017 to 629 million by 2045 [1], with approximately 80% of the people affected by diabetes residing in low- and middle-income countries (LMIC). These countries are already burdened by infectious diseases and scarce human and financial resources [2], emphasizing the importance of contextually appropriate and timely treatment of diabetes in these communities.

The importance of glycemic control in preventing and delaying the progression of diabetes complications is well established [3–5]. Indeed, the last decade has experienced considerable efforts undertaken in introducing new classes of glucose-lowering medications and formulating guidelines for the use of these therapies to optimize glycemic control [6]. However, insulin therapy remains the most widely relied upon as the mainstay therapy for diabetes [7].

Current trends on glycemic control look at several composite glycemic end points rather than individual itemized goals of measured glucose levels. This has given rise to the concept of glycemic pentads and glycemic hexads [8].

## Glycemic Control in Diabetes

### Glycemic Hexads

The terms *glycemic pentads* and *glycemic hexads* have been introduced to explain the importance of safely achieving tight glucose control [8]. The efficacy and safety objectives of the pharmacologic intervention in diabetes management need to consider the individual patient needs, fears, and comorbidity factors among others. The concept of glycemic hexads includes three efficacy parameters, namely glycosylated hemoglobin A1c (HbA1c), fasting plasma glucose (FPG), and postprandial plasma glucose (PPG), along with three safety parameters, namely hypoglycemia in general, nocturnal hypoglycemia (in special situations), and glycemic variability. Nocturnal hypoglycemia is reported as an episode of abnormally low blood glucose (3.5 mmol/L) occurring at nighttime during sleep, especially in patients with type 1 diabetes mellitus (T1DM). In the 4 years of follow-up after the Diabetes Control and Complications Trial (DCCT), 43% of all hypoglycemic episodes and 55% of severe episodes were reported to occur during sleep [9]. Patients with type 2 diabetes mellitus (T2DM) treated with long-acting sulfonylureas (SUs), insulin, or a combination of both are also susceptible to nocturnal hypoglycemia. Glycemic variability is a surrogate that explains the association between hyperglycemia and increased cardiovascular (CV) risk in persons affected by diabetes. Efforts should be made to minimize glycemic variability so as to prevent future CV events [8].

### Target Values for Glycemic Control

The primary objective in the management of diabetes is to reduce high blood glucose levels sufficiently to relieve any symptoms of hyperglycemia and to prevent/delay the onset of diabetes complications. Several surrogate markers for this important outcome have been studied: FPG, 2-h PPG, fructosamine measurements, glycated albumin, and HbA1c [10]. The HbA1c is a good surrogate marker for the long-term glycemic control [10]. In T2DM with elevated blood glucose level, high HbA1c at the time of presentation predicts a significantly increased risk of microvascular and macrovascular diseases [11, 12].

Intensive glycemic control with HbA1c target level of 6.4–7.1% (46–54 mmol/mol) is associated with reduction in risk of microvascular disease as reported by landmark trials: United Kingdom Prospective Diabetes Study (UKPDS) [4], the Kumamoto study [13], Action in Diabetes and Vascular Disease: Preterax and Diamicron MR Controlled Evaluation (ADVANCE) [14], Action to Control Cardiovascular Risk in Diabetes (ACCORD) [15], and Veterans Affairs Diabetes Trial (VADT) [16]. However, the benefits of intensive therapy should be weighed against the increase in total and cardiovascular disease (CVD)-related mortality, increased weight gain, and high risk for severe hypoglycemia [15].

The recommended HbA1c target in most patients with diabetes is < 7% (< 53 mmol/mol); in newly diagnosed patients with diabetes it is < 6.5% (< 48 mmol/mol), and in patients with diabetes who are weak with multiple comorbidities, including CVD and advanced renal disease and high hypoglycemic unawareness, it is between 7.1% (54 mmol/mol) and 8.5% (69 mmol/mol). For palliative care, the aim is to avoid symptomatic hyperglycemia.

The American Association of Clinical Endocrinologists and American College of Endocrinology (AACE/ACE) recommend an FPG target of 5.5–6.9 mmol/L [17]. It has been found that in nondiabetic individuals, the peak PPG generally does not exceed 7.8 mmol/L. However, a linear progression exists between the postload glucose level and CVD with no “lower” limit cutoff [18]. The IDF recommends a PPG target of < 9.0 mmol/L whereas the American Diabetes Association (ADA) recommends a PPG target of < 10 mmol/L [10]. Overall, the glycemic control is dependent on the HbA1c target. It is recommended in patients with stable glycemic control that HbA1c needs to be monitored at least every 6 months, while in patients who are not at target and in whom interventions have intensified, HbA1c needs monitoring at 3-month intervals.

### Recommendation for Target Values in Type 1 Diabetes Mellitus

- The target glycated hemoglobin A1c (HbA1c) for all children with type 1 diabetes mellitus (T1DM), including preschool children, is recommended to be < 7.5% (< 58 mmol/mol) **(Grade B, EL I)**

### Long-Term Glycemic and Metabolic Control

Early in the 1930s, in the first textbook on diabetes, *The Treatment of Diabetes Mellitus*, Dr. Elliot Proctor Joslin, MD advocated that diabetes should be diagnosed early and the condition should be treated vigorously through the use of carbohydrate-restricted diets and fasting and regular exercise [19]. Since then, there has been an enormous body of evidence showing that early management of diabetes with tight glucose control, i.e., keeping blood glucose levels as close to normal as possible, is vital in the prevention of short- and long-term complications of diabetes. In short, Dr. Elliot was advocating for the *Hit Early and Hit Hard* paradigm shift in the management of diabetes. In East African discourse, this would be hit early and hit hard, like hitting a snake at first sight. There are, however, still areas of controversy regarding which tools to use in achieving the tight glycemic control, and in how tight is tight enough [9, 14, 18, 20]. The following section explores the evidence available in the various approaches and how they can be fully utilized in the East African context against the background that the prevalence of diabetes and hypertension has increased in spite of a relatively low prevalence of dyslipidemia and obesity in this population [21].

### Recommendations for Target Values in Nonpregnant Adults with Type 2 Diabetes Mellitus (T2DM)

- **Fasting plasma glucose (FPG)**: 4.4–7.2 mmol/L
- **Postprandial plasma glucose (PPG)**: < 10.0 mmol/L
- **2-h postprandial plasma glucose (2-h PPG)**: < 7.8 mmol/L
- **HbA1c**:
- < 6.5% (< 48 mmol/mol) in newly diagnosed patients with T2DM; those treated with lifestyle or metformin only; T2DM with long life expectancy or no significant cardiovascular (CV) disease.
- < 7.0% (< 53 mmol/mol) in most DM patients.
- < 8.0% (< 64 mmol/mol) in patients with history of severe hypoglycemia; limited life expectancy; advanced microvascular or macrovascular complications; or long-standing diabetes in whom the goal is difficult to achieve despite diabetes self-management education, appropriate glucose monitoring, and effective doses of multiple antihyperglycemic therapy including insulin.
- For palliative care, the aim is to avoid symptomatic hyperglycemia **(Grade A, EL I)**
- **Hypoglycemia/nocturnal hypoglycemia/glycemic variability**: Achieving glycemic targets while minimizing glucose variability and hypoglycemia, particularly major and nocturnal hypoglycemia, is of much importance while considering insulin therapy **(Grade A, EL I)**

Studies from Tanzania indicate a high morbidity and mortality associated with diabetes in the region in 2017 [1]. This may be a consequence of the health system being overburdened by infectious diseases, including human immunodeficiency virus infection and acquired immune deficiency syndrome (HIV/AIDS), tuberculosis (TB), and malaria. There are significant challenges in accessing diagnosis and treatment for diabetes, further complicated by social, cultural, and ethnic factors.

In general, diabetes in East Africa is characterized by a significant number of affected individuals remaining undiagnosed, and when the diagnosis is made, it is with late presentation with the majority subsequently not accessing appropriate care [1, 22–30]. Hence, complications of diabetes are common and increasingly more patients require insulin therapy. In addition, there are challenges of accessing medicines including insulin and associated technologies [31].

Socio-politico-economic realities present challenges to individualized care in most parts of East Africa. These challenges include poverty of the population, low national spending on health, significant out-of-pocket health expenditures, medication stock-outs, and lack of facilities and equipment [32–34].

### Current Status of Diabetes Management in East Africa

The first description of diabetes in East Africa is attributed to Sir A.R. Cook [35], who, in 1901, reported that diabetes was rare in Uganda, but when encountered, it was fatal. A century later, diabetes is no longer rare, but is still fatal. Presentation of T2DM in East Africa is still characterized by acute complications superimposed on late chronic complications of diabetes, resulting mainly from delay in accessing health care [36]. Recent added challenges in the management of diabetes include comorbidity with HIV, tuberculosis, malaria, and depression. T1DM and other forms of diabetes have a similar landscape. T1DM has previously been reported as rare, probably because of being misdiagnosed. Current data on T1DM reveals an increasing incidence and prevalence, and unfortunately with high rates of complications and premature mortality [37–39].

#### Inpatient Settings in East Africa

Hospitalization for hyperglycemia is often associated with prolonged stay, rehospitalization, and increased mortality [29]. Challenges with in-hospital hyperglycemia (IHH) management include lack of trained staff, cultural practices (such as not taking *Western* medicine for abscesses), beliefs in witchcraft, and traditional medicines [40]. The majority would have diabetes just detected or would have failed on oral hypoglycemic agents or stopped medications. Insulin therapy is required for most of these patients.

#### Ambulatory Patients (Outpatient Services) in East Africa

In East Africa, ambulatory patients receive a less intensive treatment protocol for hyperglycemia with delayed insulin treatment because of multiple barriers that include availability of insulin and associated delivery devices; patients’ fear regarding insulin therapy; and physicians’ concerns for the management of primary pathology, lack of monitoring devices, poor knowledge in medical nutritional therapy, and fear of hypoglycemia [41].

#### Critical Care in East Africa

The majority of health care facilities in East Africa do not have intensive care units (ICU). Very ill patients with diabetes are managed in high dependency units or, as in most cases, general wards. The majority of this category of patients require insulin therapy.

#### Diabetes Complications in East Africa

In East Africa, short- and long-term complications of diabetes are very frequently encountered [38, 39, 42–45]. This has been attributed to multiple causes, ranging from late presentation to unavailability of services and cultural practices [29]. A significant number of patients present with long-term complications at diagnosis including diabetic foot ulcers leading to amputation [46].

## Insulin Overview

### Normal Insulin Physiology

**Fig. 1:**
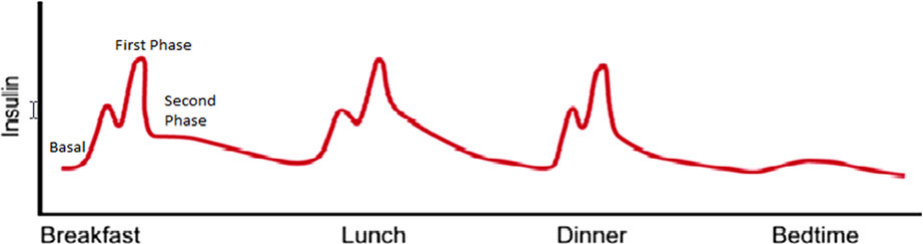
Phases of normal insulin secretion Modified from [49]

In healthy individuals, plasma glucose concentrations keep within a narrow range of about 3.5–7.0 mmol/L throughout the day despite wide fluctuations in nutritional intake, physical exercise, and other physiological, psychological, and iatrogenic determinants of plasma glucose concentrations. After food intake, plasma glucose rises to a peak in 30–60 min and returns to basal or below basal concentrations within 2–3 h. In healthy individuals, this is achieved by an appropriate response of insulin production from the β-cells of the pancreas. Approximately 50% of the total daily insulin is secreted during basal periods, suppressing lipolysis and glycogenolysis. The remainder of insulin secretion is postprandial. In response to a meal, there is a rapid and sizable release of preformed insulin from storage granules within the β-cell. This is referred to as the first phase of insulin secretion [47]. This first phase of insulin secretion promotes peripheral utilization of the prandial nutrient load, suppresses hepatic glucose production, and limits postprandial glucose elevation [48, 49]. The first phase of insulin secretion begins within 2 min of nutrient ingestion and continues for 10–15 min, giving way to the second phase of insulin secretion. The second phase of prandial insulin secretion is sustained until normoglycemia is restored. This is pictorially shown in Fig. 1. It is the loss of β-cells that underlies type 1 diabetes mellitus, and loss of β-cell glucose sensitivity and responsiveness that underlies the pathogenesis of T2DM. Between the β-cell loss and ineffective insulin release and function lie other forms of diabetes.

### Guidelines on Insulin Therapy

#### General Objectives

To provide guidelines for clinical practice on the use of insulin in diabetes based on the best available evidence to health care workers; and for the rational use of resources in the diagnosis, treatment, and follow-up of diabetes in the East African population with diabetes.

#### Specific Objectives

To improve the rational use of insulin therapy in persons presenting with diabetes who require insulin treatment.

To indicate the appropriate pharmacological interventions in the context of multifactorial treatment plan, emphasizing cost-effectiveness, individualized goals, and safety.

Key areas in insulin therapy discussed in the guidelines include insulin regimen in patients with T1DM, a stepwise approach to insulin initiation, titration, and intensification in patients with T2DM, self-monitoring of blood glucose (SMBG), awareness and management of hypoglycemia, weight gain with intensive insulin therapy, and the role of psychosocial aspects associated with insulin therapy in diabetes care.

Areas beyond insulin therapy, but within the context of improving diabetes care, that have been included in the guidelines are lifestyle modifications and target values for glucose control based on the glycemic hexads (HbA1c, FPG, PPG, hypoglycemia, nocturnal hypoglycemia, and glycemic variability) [8]. The guidelines have gone further to include a strategic treatment approach to special populations such as diabetes in pregnancy; diabetes and lactation; diabetes and renal, cardiac, and hepatic impairment; and monogenic diabetes. A discussion on the management of diabetes in special situations like Ramadan and other faith-based fasting has been included. Furthermore, diabetes management during acute and chronic infections has been included since infections may be associated with adverse outcomes in diabetes management.

## Methodology and Evidence

**Fig. 2:**
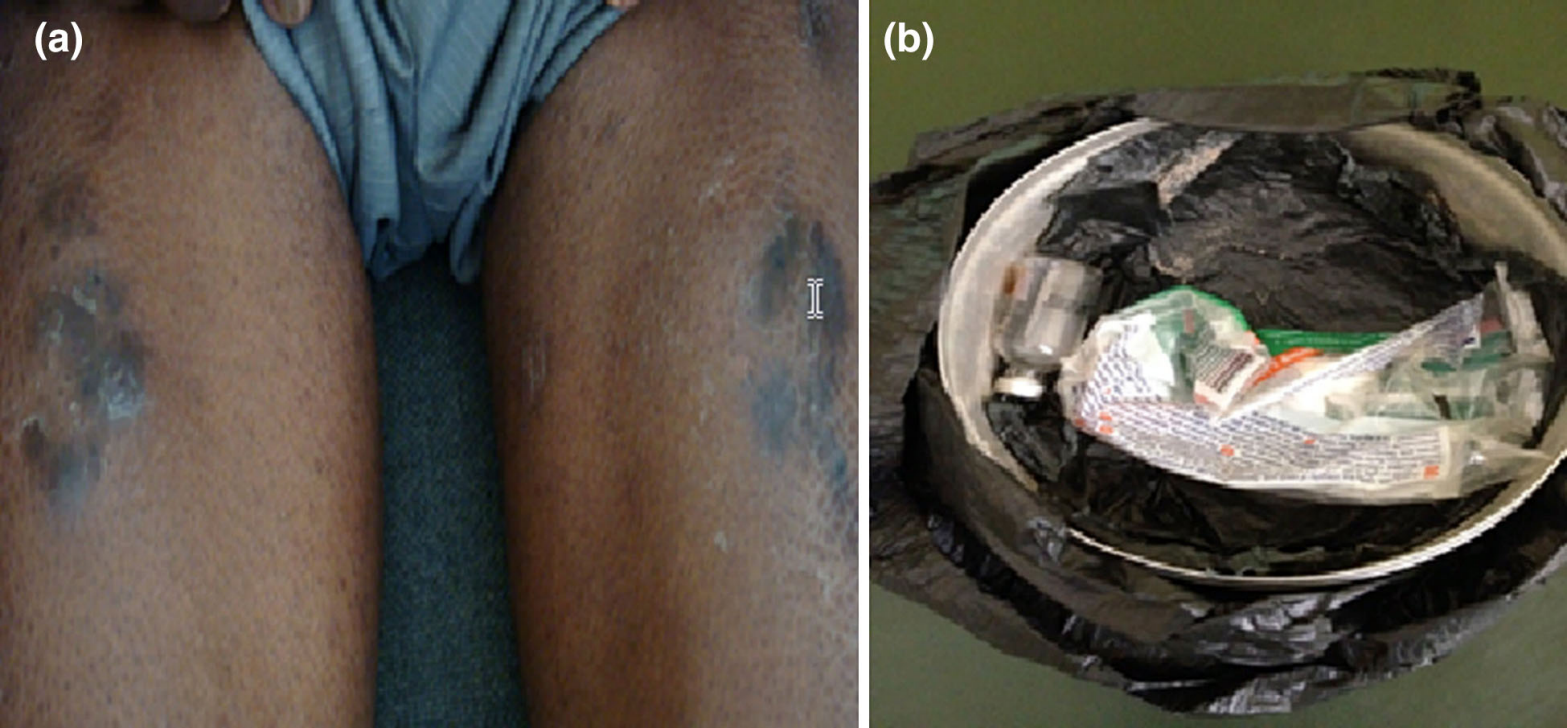
Insulin injection *tattoos* (**a**) formed as a result of overslanting the needle and injecting contaminated insulin resulting from storing it in a water container (**b**) Image courtesy of Silver Bahendeka

In drawing up the *East African Diabetes Study Group* (*EADSG) Guidelines: Insulin Therapy in Diabetes,* the authors adhered to the international and ethical standards for developing clinical practice guidelines (CPG) [41, 50]. In compliance with the Uganda National Council of Science and Technology, the photo shown in Fig. 2 was taken with the written consent of the patient embedded in his clinical notes and is anonymized. This guideline is based on previously conducted studies and does not contain any studies with human participants or animals performed by any of the authors. A systematic review of existing guidelines and select literature from medical databases (MEDLINE) and African Journals Online (AJOL) for relevant abstracts on insulin therapy was performed. The search used the medical subject headings (MeSH) terms “Insulin”, “Rapid-Acting Insulin”, “Short-Acting Insulin”, “Intermediate-Acting Insulin”, “Long-Acting Insulin”, “Beef Insulin”, “Pork Insulin”, “Insulin analogues”, “East Africa”, “Burundi”, “Kenya”, “Rwanda”, “Tanzania”, and “Uganda” in English language without date restrictions. Abstracts of all the eligible papers in English were reviewed independently and articles were considered for inclusion if they met the following criteria: meta-analysis, systematic reviews, randomized controlled trials (RCT) [phase I–IV], case reports/series, and expert opinion in the management of diabetes. Exclusion criteria were (1) studies that are not published in English language and (2) studies that did not have full papers.

The recommendations for lifestyle modification, target values for glucose control, glycemic hexads targets, stepwise approach such as insulin initiation, titration, and intensification in diabetes patients, SMBG, hypoglycemia, weight gain, and psychosocial aspects, and recommendations in special populations and special situations in Eastern Africa regions were presented, debated, and appraised by the EADSG Guidelines Development Task Force at two meetings held in November 2017 and January 2018 and based on the current global guidelines [10, 51–55].

Indications for the initiation of insulin therapy and the type of insulin to be used (human vs analogues) along with advantages and disadvantages of each insulin regimen were extensively discussed. Where there was insufficient evidence, the EADSG Guidelines Development Task Force resorted to an evidence-based consensus to arrive at the guideline. The guidelines were drafted and circulated for further review by the group members and other external reviewers within and outside Africa. The *EADSG Guidelines: Insulin Therapy in Diabetes* document was finalized as a simple, unbiased, and relevant guideline for the Eastern African settings to ensure patient values as a base for all clinical decisions.

The hierarchical system of classifying the evidence is used in these guidelines [56] where applicable, including the levels of evidence (LoE) (Table 1) and grade practice recommendations (Table 2).

**Table 1:** Level of evidence

Level	Type of evidence
IA	Systematic review (with homogeneity) of RCTs
IB	Individual RCT (with narrow CI)
IC	All or none RCT
IIA	Systematic review (with homogeneity) of cohort studies
IIB	Individual cohort study (including low quality RCT, e.g., < 80% of follow-up)
IIC	“Outcomes” research; ecological studies
IIIA	Systematic review (with homogeneity) of case–control studies
IIIB	Individual case–control study
IV	Case series (poor quality cohort and case–control study)
V	Expert opinion without explicit critical appraisal or based on physiological bench research or “first principles”

*RCT* randomized controlled trial, *CI* confidence interval

**Table 2:** Grade practice recommendations

Grade	Descriptor	Quantifying evidence	Implications for practice
A	Strong recommendation	Level I evidence or consistent findings from multiple studies of levels II, III, or IV	Clinicians should follow a strong recommendation unless a clear and compelling rationale for an alternative approach is present
B	Recommendation	Levels II, III, or IV evidence and findings are generally consistent	Generally, clinicians should follow a recommendation but should remain alert to a new information and sensitive to patient preferences
C	Option	Levels II, III, or IV evidence but findings are inconsistent	Clinicians should be flexible in their decision-making regarding appropriate practice, although they may set bounds on alternatives; patient preference should have a substantial influencing role
D	Option	Level V evidence: little or no systematic empirical evidence	Clinicians should consider all options in their decision-making and be alert to new published evidence that clarifies the balance of benefit versus harm; patient preference should have a substantial influencing role

### Insulin Therapy

Soon after the discovery of insulin in the early 1920s, both patients and health workers looked at this as a step towards the *cure* of diabetes, despite that insulin was a replacement therapy with no curative effects on the chronic state of diabetes [57]. This desire for a *cure of diabetes* is still a concern for many patients with diabetes; and for many of them, insulin therapy belongs to the category of *just a Band*-*Aid solution*. This, unfortunately, impacts negatively on insulin therapy. In spite of this, insulin therapy over the years has been revolutionized, leading to new improved formulations on the market, and devices to administer and monitor its effect [58]. Table 3 displays insulins available in East Africa. Some insulin analogues are available in pharmacies in East Africa, but are not yet on the purchase lists of governments; patients have therefore to pay out-of-pocket to access them. Moreover, their use worldwide is associated with increased cost of managing diabetes and they are thus further discouraged in low-income areas like East African countries [59]. Consequently there is no subsidy on these insulins and the full cost has to be borne by the patient. There is no published data on insurance with regards to insulin prescribing in East Africa but information obtained from dispensing pharmacies indicates that insurance companies do cover the costs when insulin analogues are prescribed.

**Table 3:** Insulins available in East Africa

Generic	Brand	Manufacturer	Form	Onset	Peak	Duration	Availability delivery	Storage
NPH	Insulatard	Novo Nordisk	Human	1–3 h	4–8 h	12–16 h	10 mL vial, 3 mL penfill (5/box)	Refrigerate: 2–8 °C, use within 6 weeks if below 25 °C, and 4 weeks if below 30 °C
	Insugen N	Biocon						
	Humulin N	Eli Lilly						
	Wosulin N	Wockhardt						
	Biosulin L	MJ Biopharm						
Regular	Actrapid	Novo Nordisk	Human	30–60 min	2–4 h	5–8 h	10 mL vial, 3 mL penfill (5/box)	Refrigerate: 2–8 °C, use within 6 weeks if below 25 °C, and 4 weeks if below 30 °C
	Insugen R	Biocon						
	Humulin R	Eli Lilly						
	Wosulin R	Wockhardt						
	Biosulin R	MJ Biopharm						
Lispro	Humalog	Eli Lilly	Analogue	10–20 min	30–90 min	3–5 h	1 × 5 × 3 mL prefilled pen	
Aspart	Novo Rapid	Novo Nordisk	Analogue	10–20 min	30–90 min	3–5 h	1 × 5 × 3 mL prefilled pen	
Glargine	Lantus	Sanofi	Analogue	60–90 min	No peak (8–12 h not pronounced)	20–26 h	1 × 5 × 3 mL prefilled pen	
	Basolog	Biocon					1 × 10 mL/1 × 3 mL vials	
Detemir	Levemir	Novo Nordisk	Analogue	60–90 min	20–26 (17.5 h reported)		1 × 5 × 3 mL prefilled pen	
Degludec	Tresiba	Novo Nordisk	Analogue	30–90 min	No peak	42 h	1 × 5 × 3 mL prefilled pen	No refrigeration for 48 days
Premixed NPH/Regular	Mixtard 30/70	Novo Nordisk	Human	Dual-acting 30–60 min	Varies maximum effect 2–8 h	10–16 h	1 × 10 mL vials and 1 × 5 × 3 mL prefilled pens	Refrigerate: 2–8 °C, use within 6 weeks if below 25 °C, and 4 weeks if below 30 °C
	Insugen 30/70	Biocon	Human					
	Humalog Mix 25	Eli Lilly	Human					
	Humalog Mix 50	Eli Lilly	Human					
	Wosulin 30/70	Wockhardt	Human					
	Insuman Combo 30	Sanofi	Human					
Aspart	NovoMix 30 (70% Protamine/30% Aspart)	Novo Nordisk	Analogue	5–15 min	Varies; ~ 1 to 4 h	10–16 h	1 × 5 × 3 mL prefilled pens	Store below 30 °C

Adopted from Uganda National Drug Authority (http://nda.or.ug/ug/register/3/Drug-Register.html), Kenya Pharmacy and Poisons Board (http://pharmacyboardkenya.org), Tanzania Food and Drug Authority (https://tfda.go.tz/portal/registered-products), Rwanda Ministry of Health (http://www.moh.gov.rw/fileadmin/user_upload/AUTHORIZED_MEDICINES_AUGUST_2017.pdf), and product leaflets

*NPH* neutral protamine Hagedorn

### Recommendation

- Insulin analogues are available in East Africa and may be safely prescribed where appropriate, taking into consideration the cost and the benefits gained when compared with human insulin that is cheaper and readily available **(Grade D, EL V)**

Various terminologies relating to the use of insulin have been used: *augmentation therapy* refers to the addition of basal insulin to a regimen when there is still some β-cell function present while *replacement therapy* refers to the use of a regimen that mimics the normal physiology of insulin secretion and is required when there is β-cell exhaustion. *Rescue therapy* refers to the use of replacement regimens for several weeks usually to reverse glucose toxicity [60]. Insulin regimens are difficult to classify, and for future comparisons of regimes of insulin in East Africa, we suggest to adopt the classification proposed by Neu et al. [61], which is based on three categories: (1) fixed insulin dose regimens, (2) glucose and meal-adjusted regimens, and (3) pump therapy. Insulins that may be used in the regimens include short-acting insulin, basal (long-acting) insulin, and premixed insulin. Blood glucose monitoring is an integral part of insulin therapy and it guides the regimens [62]. Measuring and recording both fasting and 1- or 2-h PPG levels over a 2- to 3-day period is the first step in pattern management. The patient’s insulin intake is determined by the pattern of these values, with adjustments made for anticipated need.

All patients on insulin therapy together with their caregivers require appropriate education on insulin therapy. Five basic principles of insulin management have been advised [62]: (1) insulin doses should not be skipped. Therefore to avoid high blood glucose levels caused by low or missed doses, short-acting insulin should be given every 6 h, in four equal doses, or rapid-acting insulin before each meal with a long-acting basal insulin; (2) routine daily regimens should reflect the pattern of PPG levels over the previous 2 or 3 days; (3) rapid-acting insulin doses should be based primarily on the amount to be eaten, rather than on premeal glucose levels (although abnormally elevated or depressed levels may require correction); (4) parameters for glucose levels should be set and patients instructed to call (or to administer a correction dose) if the value falls above or below a predetermined range; and (5) to consider providing patients on insulin therapy (or their parents, school nurse, hospital staff, or other health care worker) with an algorithm that uses a basal insulin dose and premeal rapid-acting insulin doses, adjusted for caloric or carbohydrate intake.

### Short-Acting Insulin

Short-acting insulin is also commonly referred to as Regular or Neutral insulin. After a subcutaneous (SC) injection, the onset of action of Regular insulin is about 30–60 min, peak effect is in 2–4 h, and duration of action is of 4–8 h [47, 63]. The larger the dose of Regular insulin, the faster the onset of action, but the longer the time to peak effect and the longer the duration of the effect. U-100 Regular insulin is absorbed slightly more quickly than U-500. Regular insulin is metabolized in the liver, spleen, kidney, and muscle. The half-life is 30–60 min [64]. Without using other forms of insulin, short-acting insulin should be given every 6 h, in four equal doses, without ever skipping a dose. Details of pharmacodynamics of short-acting insulin are displayed in Table 3.

### Rapid-Acting Insulin

Rapid-acting insulins are typically insulin analogues that were developed to better control excursions of blood glucose following a meal ingestion, by achieving a pharmacokinetic profile more similar to mealtime endogenous insulin than human unmodified insulin does [65]. In East Africa the three commonly encountered rapid-acting insulin analogues are insulin lispro (Humalog^®^; Eli Lilly, Indianapolis, IN, USA), insulin aspart (Novolog^®^/NovoRapid^®^; Novo Nordisk, Bagsvaerd, Denmark), and insulin glulisine (Apindra^®^; Sanofi, Paris, France). Rapid-acting insulin should be injected before each meal in three daily doses if the patient is also taking a long-acting or intermediate-acting insulin, or six times a day if used without basal insulin. Rapid-acting insulins are only available in pharmacies located in the main towns of East Africa, and should be used taking into consideration the cost and the benefits gained when compared with human insulin that is cheaper and readily available.

### Basal (Long-Acting) Insulin

Insulin is secreted at a low (basal) level in the non-fasted state, with increased, stimulated levels at mealtimes [49]. Several exogenous long-acting insulin formulations are now available in the market. In East Africa, the available long-acting insulins include neutral protamine Hagedorn (NPH), insulin glargine U-100, insulin glargine U-300, insulin detemir, and insulin degludec. They mainly vary in their duration of action and peak effect. The lower the peak, the lower the risk of hypoglycemia.

Compared with NPH insulin, insulin glargine shows a flatter pharmacologic profile with no pronounced peak and longer duration of action of about 24 h. Within-subject variability has been shown to be lower with insulin glargine relative to NPH insulin [49]. Results from a meta-analysis of clinical trials show that among all the basal insulins, insulin degludec with ultra-long duration of action exhibits a greater reduction of HbA1c, least variability of action, and lowest rate of hypoglycemia [66]. In persons prone to hypoglycemia, insulin degludec is the preferred basal insulin. However, the higher cost of this insulin and its unavailability in pharmacies outside main towns in East Africa make the recommendation for its use in the region to be highly individualized.

### Premixed Insulins

Premixed insulins are short-acting insulin (Regular/Neutral) or rapid-acting analogue insulin mixed with intermediate-acting insulin in a fixed ratio, addressing both FPG and PPG in a single injection (biphasic human insulin [30/70, 50/50], biphasic insulin aspart [30/70, 50/50], and biphasic insulin lispro [25/75, 50/50]). Those available in East Africa are shown in Table 3. The perceived advantages of using premixed insulin over a self-mixed insulin include the increased accuracy of dosage, efficacy, and patient convenience, which may translate to increased compliance and thus better long-term control of diabetes [67, 68].

## Insulin Therapy in T1DM

In the absence of obesity, all patients less than 30 years old should be treated as for T1DM unless they are less than 1 year old, have no ketones, have optic atrophy, retinitis pigmentosa, deafness, or other systemic illness [69]. T1DM is treated with insulin [70]. Treatment focuses on preventing complications by managing blood glucose levels with insulin, diet, and lifestyle modification [71, 72]. Multiple daily injections of short-acting or rapid-acting insulin analogues, given 0–15 min before meals together with one or more daily separate injections of intermediate or long-acting insulins, are used. The basal-bolus regimen includes basal insulin (insulin degludec, insulin glargine, insulin detemir, and NPH) and bolus insulin (rapid-acting: insulin aspart, insulin lispro, or insulin glulisine; or short-acting: Regular/Neutral). Patients with severe decompensation (e.g., diabetic ketoacidosis, DKA) require intensive therapy, usually using short-acting insulin under close supervision [73]. In the DCCT study in which short-acting and intermediate-acting human insulins were used, intensive therapy with multiple daily injections or continuous subcutaneous insulin infusion (CSII) improved glycemia and resulted in better long-term outcomes [74].

The basal-bolus regimen showed improved PPG control and less hypoglycemia when compared with Regular insulin. Preprandial administration of insulin glulisine or insulin lispro showed better glycemic control [75]. Insulin aspart has been associated with improved quality of life (QOL). In patients with good glycemic control, insulin detemir and insulin glargine (with Regular insulin or bolus insulin) lowered FPG with less nocturnal hypoglycemia when compared with once- or twice-daily NPH insulin [76]. Improved QOL was reported in patients with use of insulin glargine when compared with use of NPH in a basal-bolus insulin regimen [77]. The preinjection hyperglycemia in T1DM with insulin glargine can be prevented by twice-daily administration of the insulin. Twice-daily insulin detemir in a basal-bolus regimen showed less nocturnal hypoglycemia and improved glycemic control in several studies [78]. An ultra-long-acting insulin analogue, insulin degludec, in T1DM showed comparable safety and tolerability and less hypoglycemia when compared with insulin glargine [79]. As a result of their inflexible timing, long-acting analogue insulins may lead to hypoglycemia. The disadvantages of inflexibility with long-acting analogue insulin may be addressed with the use of modern insulin pump therapy. Evidence showed that premixed insulin analogues resulted in significant reduction in HbA1c levels [80] and similar safety profile [81] when compared with human premixed insulins. Moreover, premixed insulin analogues resulted in a better PPG control when compared with premixed human insulin.

Pramlintide, an amylin analogue, works by delaying gastric emptying, blunts pancreatic secretion of glucagon, and enhances satiety in T1DM. It has been shown to induce weight loss and lower insulin doses in T1DM. Pramlintide is not available in East Africa. For the management of T1DM in obese patients, the use of metformin reduces the insulin requirements and the total cholesterol/low-density lipoprotein (LDL) ratio with less weight gain [82].

### Recommendations

- Basal-bolus insulin therapy is a standard of care in management of diabetes in T1DM **(Grade A, EL I)**
- For T1DM patients with minimal metabolic decompensation (minimal dehydration, fully conscious) initiation starts with initial dose ranging from 0.4 to 1.0 units/kg/day **(Grade A, EL III)**
- Multiple daily injections of short-acting or rapid-acting insulin analogues given 0–15 min before meals together with one or more daily separate injections of intermediate or long-acting insulins are used. The basal-bolus regimen includes basal insulin (insulin degludec, insulin glargine, insulin detemir, and NPH) and bolus insulin (rapid-acting: insulin aspart, insulin lispro, or insulin glulisine; or short-acting: Regular/Neutral) **(Grade A, EL I)**
- Consider using rapid-acting insulin analogues for less hypoglycemia risk **(Grade B, EL I)**
- If other insulins are not available, premixed insulin injections may be used in T1DM adolescent patients. Premixed insulin analogues should be considered over human insulin for favorable degree of PPG control and significant lowering of HbA1c **(Grade C, EL III)**
- Patient education on matching prandial insulin doses to carbohydrate intake, premeal blood glucose levels, and anticipated physical activity should be encouraged **(Grade A, EL II)**

## Type 2 Diabetes Mellitus (T2DM)

T2DM is a progressive disease leading to oral hypoglycemic failure and subsequent requirement for insulin therapy. Therefore, the key concept in the treatment of T2DM is establishing individualized glycemic goals based on each patient’s clinical characteristics. This individualized care influences the choice of antihyperglycemic therapy as the disease progresses over time.

Insulin is indicated in known patients with T2DM if the HbA1c level remains persistently above 10.0% (86 mmol/mol) or uncontrolled diabetes with respect to predefined goals in spite of optimizing the oral antidiabetic drugs (OADs).

The initial step would be combining OADs with basal insulin (augmentation). If this intervention does not result in the required glycemic control, then consideration should be given to change the therapy to replacement therapy with insulin, which should be intensified as appropriate for the individual.

In newly diagnosed T2DM patients who are symptomatic, insulin may be the initial therapy to stabilize the glycemia and alleviate the symptoms (rescue therapy).

**Fig. 3:**
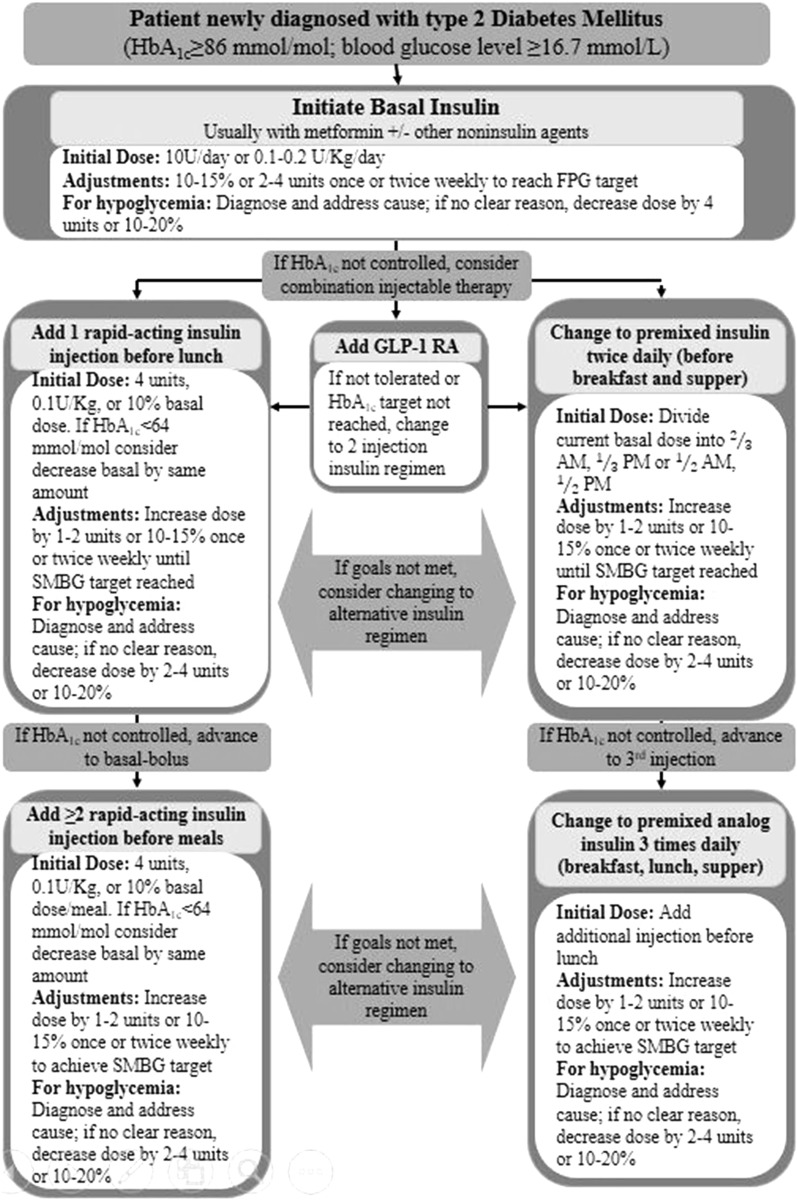
Initiation of insulin therapy with basal insulin. HbA1c glycated hemoglobin A1c, FPG fasting plasma glucose, GLP-1 RA glucagon like peptide-1 receptor agonist, SMBG self-monitoring of blood glucose Modified from [104]

An algorithm showing the OADs leading to insulin is depicted in Fig. 3.

### Insulin Therapy in T2DM: The Initiation Algorithm

Initiation of insulin therapy is preceded by a decision on the right insulin regimen, identifying the right formulation, doses, and appropriate delivery devices, and correct strategies for dose titration. The patient-centered treatment plan in the management of diabetes should focus specifically on matching the insulin supply to the regular diet/exercise patterns of diabetes patients and follow-up with regular SMBG [10, 83]. The overall strategy is to first correct FPG with a dinnertime/bedtime insulin followed by a focus on PPG. As elevated PPG levels are a substantial contributor to daytime hyperglycemia, targeting PPG control becomes vital in achieving optimal glycemic control.

Once the decision has been made to initiate insulin, clinical, pharmacological, and psychosocial factors must be considered and factored into the patients care plan. In addition, other factors such as cost of insulin, quality, cold chain management for insulin, and continuous availability of insulin preparations as well as delivery devices should be contextually discussed with the patient, family, and other caregivers.

The FPG and PPG measurements together with HbA1c value provide some information for the physician to choose an insulin type by following simple ratios: ratio of prandial and FPG index ([PPG – FPG]/FPG); a high ratio implies a higher prandial component, which will require premixed or rapid-acting/short-acting insulin, while a low level suggests a greater contribution of the fasting component of hyperglycemia and supports the use of basal insulin. The ratio of FPG to HbA1c with a cutoff 1.3 gives an indication of the contribution of fasting hyperglycemia. Serum 1,5-anhydroglucitol (1,5-AG) drops as serum glucose rises above the renal threshold and has been proposed as a marker for postprandial hyperglycemia. In clinical practice HbA1c and 1,5-AG may be used sequentially, first utilizing HbA1c to identify patients who are moderately or well controlled (HbA1c 6.5–8.0% [48–64 mmol/mol]) and then using the 1,5-AG assay to determine the extent of prandial glucose excursions [84]. Initiating insulin therapy with basal insulin is recommended by ADA 2018 and AACE/ACE [10, 17]. The IDF recommends to initiate insulin therapy with either basal or premix insulins [52]. The premix insulin analogues are preferred over human premix insulins owing to the lower incidence of severe hypoglycemia, less nocturnal hypoglycemia, and flexibility of administration [53]. The Indian National Consensus Group (INCG) 2013 recommends only premix insulin at the initiation. In addition, as rescue therapy, the INCG recommends initiation of insulin in newly diagnosed T2DM patients [54].

#### Basal (Long-Acting) Insulin Regimen

Basal insulin controls glycemia by suppressing hepatic glucose production in between meals and during sleep. The intermediate-acting NPH, long-acting (insulin glargine and insulin detemir), or ultra-long-acting (insulin degludec) formulations offer relatively uniform 24-h coverage of blood glucose levels. Evidence showed that insulin glargine, insulin detemir, and insulin degludec are associated with less overnight hypoglycemia when compared with NPH and relatively less weight gain [85]. Several comparative trials between insulin glargine, insulin detemir, and insulin degludec show varying dose requirements for effective glycemic control, and higher average unit requirement with insulin detemir compared with insulin glargine [86, 87]. Figure 3 shows the steps for initiation with basal insulin therapy.

#### Bolus Insulin Regimen

Most patients with T2DM may require mealtime bolus insulin dosing in addition to basal insulin. Rapid-acting analogues (insulin aspart, insulin lispro, or insulin glulisine) are preferred owing to their prompt onset of action after dosing. The recommended starting dose of mealtime insulin is 4 units, 0.1 units/kg, or 10% of the basal dose.

#### Premix Insulin Regimen

**Fig. 4:**
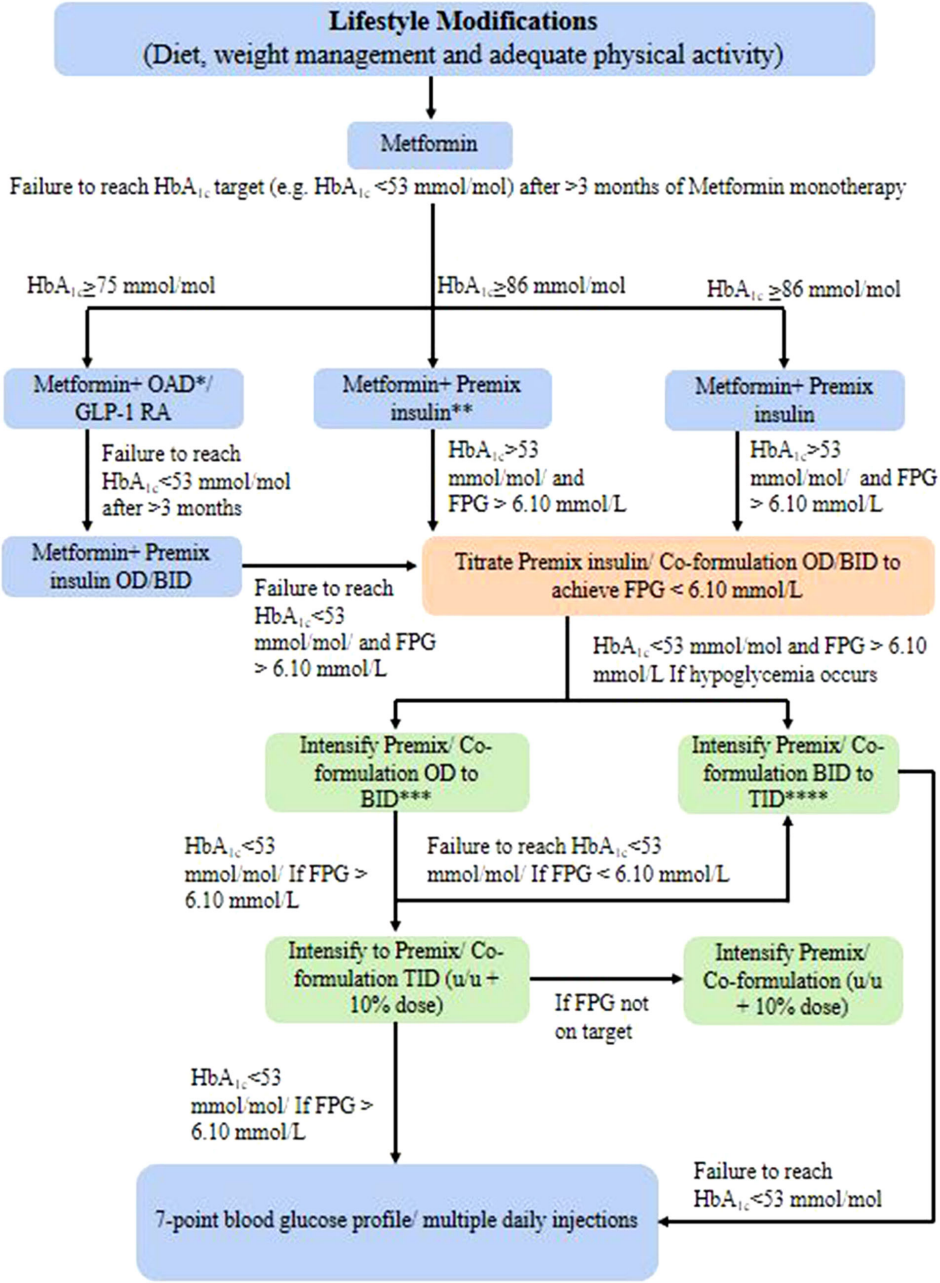
Initiation of insulin therapy with premix/insulin co-formulation. OAD oral antidiabetic agents, GLP-1 RA glucagon-like peptide-1 receptor agonist, OD once daily, BID twice daily, TID three times in a day. *OAD can be a sulfonylurea/thiazolidinedione/dipeptidyl peptidase-4 inhibitor or any other drug as per clinician’s judgment; **Start with OD 10–12 units (0.1–0.2 U/kg body weight). In the morning if the predinner blood glucose is high. In the evening if the prebreakfast blood glucose is high. Split the dose when dose is > 30 units. ***Intensification from OD to BID. Split the OD dose into equal breakfast and dinner doses (50:50). ****Intensification from BID to TID. Add 2–6 U or 10% of total daily premix dose before lunch. Down-titration of morning dose (− 2 to 4 U) may be needed after adding lunch dose. In both cases, continue metformin and administer premix just before meals Modified from [54]

Most patients with T2DM are treated with premix insulins (biphasic human insulin [30/70, 50/50], biphasic insulin aspart [30/70, 50/50], or biphasic insulin lispro [25/75, 50/50]) or insulin degludec/insulin aspart 70/30. Figure 4 shows steps for initiating premix/insulin co-formulations. Premix insulin (10 U) once daily (OD) can be started either in the morning if predinner glucose is high or at night if the prebreakfast glucose is high. If a patient on biphasic insulin aspart 30 OD or BID has within-target FPG but has an HbA1c > 7.0% (> 53 mmol/mol), a switch to biphasic insulin aspart 30 BID or TID should be considered. If their FPG is above target, the dose should be titrated to achieve FPG 4.0–6.0 mmol/L; however, if hypoglycemia occurs, an additional daily dose should be added rather than further dose titration [88]. When the daily insulin dose in OD regimen exceeds 20 U, intensify the regimen to BID such that the dose is distributed as two-thirds in the morning and one-third in the evening. However, when the single dose exceeds 30 U, the dose can be split into two equal doses, which reduces the chance of hypoglycemia. Also, the initial dose distribution ratio for morning and evening doses is 50:50% for biphasic insulin aspart 30, biphasic insulin lispro 25, and insulin degludec/insulin aspart 70/30 in case of patients with higher HbA1c or if blood glucose control is suboptimal [54]. The lower incidence of major and nocturnal hypoglycemia and flexibility of administration with premix insulin analogues have made this regimen a better choice over human premix insulins when initiating insulin therapy. However, insulin degludec/insulin aspart 70/30 may be preferred over other premix insulin analogues considering lower incidence of overall and nocturnal hypoglycemia and superior FPG control when used [53]. The advantages of premix insulin analogues and insulin co-formulations over premix human insulins are displayed in Table 4 [53].

**Table 4:** Comparisons between premixed human insulins vs premixed insulin analogues vs insulin co-formulation in patients with T2DM Modified from [54]

Parameter	Premixed human insulin	Premixed insulin analogue	Insulin co-formulation
PPG control	+	+++	+++
FPG control	++	++	+++
HbA1c control	+	++	++
Less hypoglycemia	+	++	+++
Mealtime flexibility	+	+++	+++
Weight gain	+	++	++

*PPG* postprandial glucose, *FPG* fasting plasma glucose, *HbA1c* glycated hemoglobin A1c

Recent studies seem to suggest better outcomes with insulin analogues. Results from the A1chieve study showed that T2DM initiated with premix insulin was associated with a mean change of HbA1c of 1.7% [− 18 mmol/mol] from a baseline of 9.1% [76 mmol/mol] and significantly reduced PPG in an African subgroup similar to those in the overall population [89]. In addition, switching from human insulin to premix analogues showed a similar positive safety profile. Evidence suggests that in insulin-naïve African subjects with T2DM, initiating once-daily premix insulin with or without OADs achieved better glycemic control than when compared with human insulin [90]. In a systematic review, short-term intensive insulin therapy was reported to improve the underlying pathophysiology in newly diagnosed T2DM. The study results showed that intensive insulin therapy leads to an increase in β-cell function and a decrease in insulin resistance when compared with baseline values [91].

In patients with T2DM poorly controlled on OADs with HbA1c > 9.0% (> 75 mmol/mol), initiating premix insulin analogue therapy is superior to basal insulin analogues or human premix insulin [92–99]. The initiation of premix insulin analogues is recommended in insulin-naïve patients aged more than 65 years with T2DM poorly controlled by OADs with dietary counseling interventions for improved glycemic control and significant reduction in FPG.

### Recommendations

Initiation

- Do not delay the decision to initiate insulin in diabetes patients **(Grade A, EL I)**
- Health care professionals (HCPs) should educate T2DM patients about insulin regimens and appropriate choice of regimen **(Grade A, EL III)**
- Consider initiating insulin therapy with or without metformin in patients with newly diagnosed T2DM who are symptomatic and/or have HbA1c ≥ 10.0% (≥ 86 mmol/mol) and/or blood glucose levels ≥ 16.7 mmol/L **(Grade A, EL I)**
- Initiate with once-daily basal insulin, once-daily premixed/co-formulation insulin, or twice-daily premixed insulin, either alone or in combination with GLP-1 RA (either alone or in combination with basal insulin, in same pen device) or in combination with other OADs on the basis of clinical features, glucose profile, risk of hypoglycemia, and patient preference **(Grade A, EL II)**
- Basal-bolus insulin regimens may be needed in severe hyperglycemia and in life-threatening or organ/limb-threatening clinical situations **(Grade A, EL III)**
- Analogue insulins with possible lower risk of nocturnal and symptomatic hypoglycemia may be used in preference to human insulins; however, economic considerations must be taken into account. Newer insulin co-formulations are associated with fewer hypoglycemic episodes (**Grade B, EL I)**
- Match the insulin dose to carbohydrate intake **(Grade A, EL II)**
- Counseling on scheduling regular blood glucose monitoring and awareness of hypoglycemic symptoms and their management are recommended to all patients initiating with insulin. The HCPs should provide guidance for adjusting insulin dose adjustments, administration, storage, and other practical aspects **(Grade A, EL I)**

Titration

- Target for FPG level is 4.4–7.2 mmol/L, PPG level is < 10 mmol/L, and 2-h PPG level is < 7.8 mmol/L. These targets can be individualized on the basis of the risk of hypoglycemia and the urgency for glycemic control **(Grade A, EL I)**
- Titration should be done at regular and short intervals to attain glycemic goals without causing hypoglycemia **(Grade A, EL I)**

### Intensification of Insulin Therapy

The long-term follow-up UKPDS and DCCT stressed the importance of intensive glycemic control with insulin, especially from the early stages of diagnosis of diabetes [9, 83].

#### Basal Plus/Basal-Bolus Insulin

As a result of progressively diminishing insulin secretory capacity, more patients with T2DM may require prandial insulin therapy in addition to the existing one or two doses of insulin. This is typically achieved with Regular insulin administered about 30 min before meals or rapid-acting insulin analogues such as insulin lispro, insulin aspart, or insulin glulisine, which can be injected just before or with the meal. Insulin analogues give better PPG control than human Regular insulin. Furthermore, an analogue-based basal-bolus regimen may be preferred over human basal-bolus regimen considering the significantly lower risk of nocturnal hypoglycemia and better outcomes in patients with T2DM [100]. The steps for initiation of basal therapy and intensification of insulin therapy are shown in Tables 5 and 6, respectively.

**Table 5:** Initiation of basal therapy Modified from [17]

	Glucose value	Total daily dose
Step 1: initiation with basal insulin^a^	HbA1c < 8% (< 64 mmol/mol)	0.1–0.2 units/kg
	HbA1c > 8% (> 64 mmol/mol)	0.2–0.3 units/kg
Step 2: titration^b^ (every 2–3 days to reach glycemic goals)	Fixed regimen	Increase by 2 units/day
	Adjustable regimen	
	FPG > 10 mmol/L	Add 4 units
	FPG 7.77–10 mmol/L	Add 2 units
	FPG 6.11–7.72 mmol/L	Add 1 unit
Step 3: monitor for hypoglycemia	BG < 3.88 mmol/L	Reduce by 10–20%
	BG < 2.22 mmol/L	Reduce by 20–40%

*HbA1c* glycated hemoglobin A1c, *BG* blood glucose, *FPG* fasting plasma glucose, *NPH* neutral protamine Hagedorn, *SU* sulfonylureas

^a^Consider discontinuing SU therapy and basal analogues should be preferred over NPH insulin

^b^For most patients with T2DM taking insulin, glucose goals are HbA1c < 7% (< 53 mmol/mol) and fasting and premeal blood glucose < 6.11 mmol/L in the absence of hypoglycemia. HbA1c and FPG targets may be adjusted on the basis of patients age, duration of diabetes, presence of comorbidities, diabetic complications, and hypoglycemia risk

**Table 6:** Intensification of premix/insulin co-formulation Modified from [17]

	Therapeutic option	Total daily dose
Step I: add prandial insulin	When glycemic targets are unmet	TDD 0.3–0.5 units/kg (40–50% basal: 50–60% prandial)^a^
Step II: titration^b^ (every 2–3 days to reach glycemic goals)	Fixed regimen (prandial insulin)	Increase TDD by 2 units/day
	Adjustable regimen (prandial insulin)	
	FPG > 9.99 mmol/L	Increase TDD by 4 units
	FPG 7.77–9.99 mmol/L	Increase TDD by 2 units
	FPG 6.10–7.71 mmol/L	Increase TDD by 1 unit
	2-h PPG or next premeal glucose > 9.99 mmol/L	Increase prandial dose for the next meal by 10%
	When glycemic targets are unmet	TDD 0.3–0.5 units/kg (40–50% basal: 50–60% prandial)*
	FPG/premeal BG > 9.99 mmol/L	Increase TDD by 10%
Step III: monitor for hypoglycemia	Fasting hypoglycemia	Reduce basal insulin dose
	Nighttime hypoglycemia	Reduce basal insulin or reduce short/rapid-acting insulin taken before supper or evening snack
	Between-meal hypoglycemia	Reduce previous premeal short/rapid-acting insulin

*BG* blood glucose, *DPP*-*4* dipeptidyl peptidase-4 inhibitors, *FPG* fasting plasma glucose, *GLP*-*1* glucagon-like peptide 1 receptor agonists, *NPH* neutral protamine Hagedorn, *PPG* postprandial glucose, *SGLT2* sodium glucose cotransporter 2, *TDD* total daily dose

^a^Basal + prandial insulin analogues preferred over NPH + Regular insulin or premixed insulin

^b^For most patients with T2DM taking insulin, glucose goals are HbA1c < 7% (< 53 mmol/mol) and fasting and premeal blood glucose < 6.10 mmol/L in the absence of hypoglycemia. HbA1c and FPG targets may be adjusted on the basis of patient’s age, duration of diabetes, presence of comorbidities, diabetic complications, and hypoglycemia risk

#### Premix Insulin

The INCG 2013 recommends to intensify premix insulin to twice and thrice daily if HbA1c is > 7.0% (> 53 mmol/mol) and FPG is > 6.1 mmol/L [54]. If glycemic control with premix/basal insulin is not achieved then twice-daily insulin degludec/insulin aspart 70/30 is preferred over premix insulin analogues for intensification. Furthermore, a recent systematic review suggests that in insulin-treated T2DM, insulin degludec/insulin aspart 70/30 twice daily is comparable to biphasic insulin aspart 30 twice daily and imposes a lower risk of nocturnal hypoglycemia [101]. Insulin degludec/insulin aspart 70/30 improved long-term glycemic control with greater reduction in FPG with a lower dose and less nocturnal hypoglycemia, when compared with biphasic insulin aspart 30 [102–104].

#### GLP-1 Receptor Agonists

The injectable glucagon-like peptide-1 receptor agonists (GLP-1 RAs) (liraglutide, exenatide, lixisenatide, dulaglutide, and albiglutide) mimick the effects of endogenous GLP-1, thereby stimulating pancreatic insulin secretion in a glucose-dependent fashion, suppressing pancreatic glucagon output, slowing gastric emptying, and decreasing appetite. Advantages of this regimen include significant weight loss. However, this therapy produces nausea and vomiting, particularly early in the course of treatment. Generally, GLP-1 RAs and their combinations are not available in East Africa.

#### Combination Injectable Therapy (Insulin + GLP-1 RA)

Consider a combination injectable therapy if the basal insulin has been titrated to acceptable FPG level or if the dose is 0.5 U/kg/day and HbA1c remains above the target. Advantages include less hypoglycemia risk and less weight gain. GLP-1 RAs are associated with transient gastrointestinal (GI) side effects.

### Recommendations

Intensification

- Intensification of insulin therapy should be considered when patients fail to achieve glycemic goals even after optimal dose titration **(Grade A, EL I)**
- Intensification with premix insulin twice daily or thrice daily, insulin co-formulation-based regimen, prandial insulin (basal plus or basal bolus) with the largest meal of the day, or GLP-1 RA. Choice of intensification regimen is based upon diet, lifestyle, risk of hypoglycemia and weight gain, affordability, and patient preference **(Grade A, EL II)**

GLP1-RA therapy

- In T2DM patients with uncontrolled hyperglycemia, GLP-1 RAs are suitable second-line or third-line treatment option **(Grade A, EL I)**

### Insulin in Special Populations

#### Newly Diagnosed T2DM

Insulin therapy (with or without additional agents) in newly diagnosed T2DM is preferred if HbA1c is ≥ 10.0% (≥ 86 mmol/mol), FPG > 13.9 mmol/L, PPG > 16.7 mmol/L, and/or if the patient is symptomatic. Consider initiating dual therapy in patients with newly diagnosed T2DM if HbA1c ≥ 9% (≥ 75 mmol/mol) [10]. After glycemic and metabolic control, patients may be started on oral hypoglycemic agents.

#### Recommendations

- For newly diagnosed T2DM, consider initiating insulin therapy, if HbA1c ≥ 10.0% (≥ 86 mmol/mol), FPG > 13.9 mmol/L, PPG > 16.7 mmol/L, and/or if patient is symptomatic **(Grade A, EL I)**
- For newly diagnosed T2DM, consider initiating dual therapy, if HbA1c 9.0% (≥ 75 mmol/mol) **(Grade A, EL II)**

#### Elderly

Elderly patients with diabetes are at an increased risk of hypoglycemia and therapy with a low risk of hypoglycemia should be the choice of treatment. Metformin is the first-line agent for older adults with T2DM. Use of SUs and other insulin secretagogues with high risk of hypoglycemia should be used with caution. When insulin therapy is required, most elderly patients with advanced diabetes complications, life-limiting coexisting chronic illnesses, or limited functional status, once-daily basal insulin injection therapy is preferred to multiple daily injections as the latter may be too complex for them. SMBG using home glucose meters is encouraged; patients or their caregivers are instructed on dose adjustment according to results of SMBG and steps to take for hypo- and hyperglycemic episodes [10].

#### Recommendations

- Consider antihyperglycemic therapy with low risk of hypoglycemia in elderly patients who are at increased risk of hypoglycemia **(Grade A, EL II)**
- Consider once-daily basal insulin injection regimen over multiple daily insulin injection regimen to reduce the risk of hypoglycemia if glycemic goals can be achieved within the individualized HbA1c target **(Grade B, EL II)**

#### Pregnancy

Pregnancy outcomes of mothers with diabetes during pregnancy are associated with high rates of complications in both the mother and baby. Polyhydramnios, intrauterine fetal death, macrosomia, and stillbirths are frequently reported [105–107]. Glycemic targets during pregnancy have become more stringent [108]: the HbA1c goal is 6.0–6.5% (42–48 mmol/mol)—the goal of 6.0% (42 mmol/mol) may be optimal if this can be achieved without significant hypoglycemia, but the target may be relaxed to 7.0% (53 mmol/mol) if necessary to prevent hypoglycemia; FPG 5.3 mmol/L; PPG 7.8 mmol/L; and 2-h PPG 6.7 mmol/L. Where glycemic targets have been achieved with metformin monotherapy, the use of metformin during pregnancy has been associated with a lower risk of neonatal hypoglycemia and less maternal weight gain than insulin. In the short term, in women with gestational diabetes mellitus (GDM) requiring drug treatment, glibenclamide is clearly inferior to both insulin and metformin, while metformin (plus insulin when required) performs better than insulin [109]. In most East African countries, insulin (between once and three times daily) is the treatment of choice to control hyperglycemia in GDM. Recent data suggest no significant difference between premix insulin analogues and premix human insulin in terms of glycemic control or fetal outcome (neonatal birth weight). However, premix insulin analogues offer flexible dosing and a high safety profile compared with premix human insulin [110].

#### Recommendations

- Tighter glycemic targets are suggested during pregnancy: HbA1c 6.0–6.5% (42–48 mmol/mol); FPG 5.3 mmol/L; 1-h PPG 7.8 mmol/L; and 2-h PPG 6.7 mmol/L **(Grade A, EL I)**
- Insulin is the preferred medication for treating hyperglycemia in gestational diabetes mellitus (GDM) as it does not cross the placenta **(Grade A, EL I)**
- Antenatal patients may use any human insulin preparation, insulin aspart or insulin lispro preparations, or insulin detemir (**Grade A, EL II)**

#### Recommendation

- Consider using premix insulin with proven safety profile in lactating mothers with diabetes **(Grade B, EL II)**

#### Lactation

Individualized treatment approach is advised in lactating mothers with diabetes. It is safe to treat them with premix insulin owing to their proven safety profile.

#### Renal Impairment

The overall prevalence of chronic kidney disease (CKD) in sub-Saharan Africa (SSA) is 13.9% with a wide variation between East African regions (from 30% in Zimbabwe to 2% in Côte d’Ivoire) [111]. Chronic renal failure (CRF) is associated with diverse alterations in carbohydrate and insulin metabolism. Insulin therapy with premix insulin analogues can notably improve glycemic control in CRF diabetic patients [112]; however, the choice of insulin therapy should be individualized.

#### Recommendations

- Consider using insulin analogues in renal impaired patients with diabetes for improved glycemic control with low risk of hypoglycemia **(Grade B, EL II)**
- Frequent blood glucose monitoring and dose adjustments are recommended in chronic renal failure (CRF) diabetic patients when they are treated with insulin **(Grade B, EL II)**

#### Cardiac Impairment

Coronary heart disease (CHD) affects 5–8% of diabetic patients in SSA [113]. Premix insulin analogues reduce PPG more effectively than premix human insulins and basal insulin analogues. The choice of insulin regimen should be individualized and based upon cost, severity of hyperglycemia, risk of hypoglycemia, and likelihood of interventional procedures in the very near future.

#### Recommendations

- Choice of insulin regimen and preparation should be based upon cost, severity of hyperglycemia, risk of hypoglycemia, and likelihood of interventional procedure in near future **(Grade B, EL II)**
- Patients with T2DM on combination therapy of premixed insulin analogues and OADs should be carefully monitored for signs and symptoms of heart failure (HF), weight gain and edema, and a prompt clinical action is recommended if any deterioration in cardiac symptoms occurs **(Grade B, EL II)**

#### Hepatic Impairment

The patients with T2DM are at risk of developing non-alcoholic fatty liver disease (NAFLD) and therefore patients with diabetes and hepatic impairment are likely to be encountered in East Africa [114]. When insulin therapy is required in patients with hepatic impairment, the choice should be regimens with low risk of hypoglycemia.

#### Recommendation

- Consider using insulin analogues in T2DM patients with hepatic impairment for improved glycemic control with low risk of hypoglycemia **(Grade B, EL II)**

#### Monogenic Forms of Diabetes

Mutations which may be dominantly or recessively inherited from either parent or occur as a de novo mutation in a single gene affect the functioning of the insulin-producing pancreatic β-cells and precipitate a rare form of diabetes termed as monogenic. The worldwide prevalence of the monogenic form of diabetes is estimated at 1–2% of all pediatric diabetes [115]. Nyangabyaki-Twesigye et al. reported the first case in East Africa with permanent neonatal diabetes due to a mutation in the *KCNJ11* gene encoding the Kir6.2 subunits in a 6-month-old subject [116]. The monogenic form of diabetes can be differentiated as transient and permanent neonatal diabetes. In both conditions, hyperglycemia is a common phenomenon. In the transient type, the patient may recover spontaneously at 3 months with no further requirement for insulin. The International Society for Pediatric and Adolescent Diabetes (ISPAD) recommends genetic testing for diagnosis of this rare form of diabetes for optimal treatment [117]. Although such tests are expensive and not readily available in East Africa, some centers outside East Africa offer them, for research purposes. In most patients with permanent neonatal diabetes, lifelong insulin therapy is required, and management follows guidelines for T1DM [118]. In children with an ATP-sensitive potassium channel defect in the pancreatic β-cells, treatment with oral high dose glibenclamide is an optional therapy.

#### Recommendation

- In most patients with permanent neonatal diabetes, lifelong insulin therapy is required **(Grade A, EL III)**

#### Ramadan and Other Fasting States

**Fig. 5:**
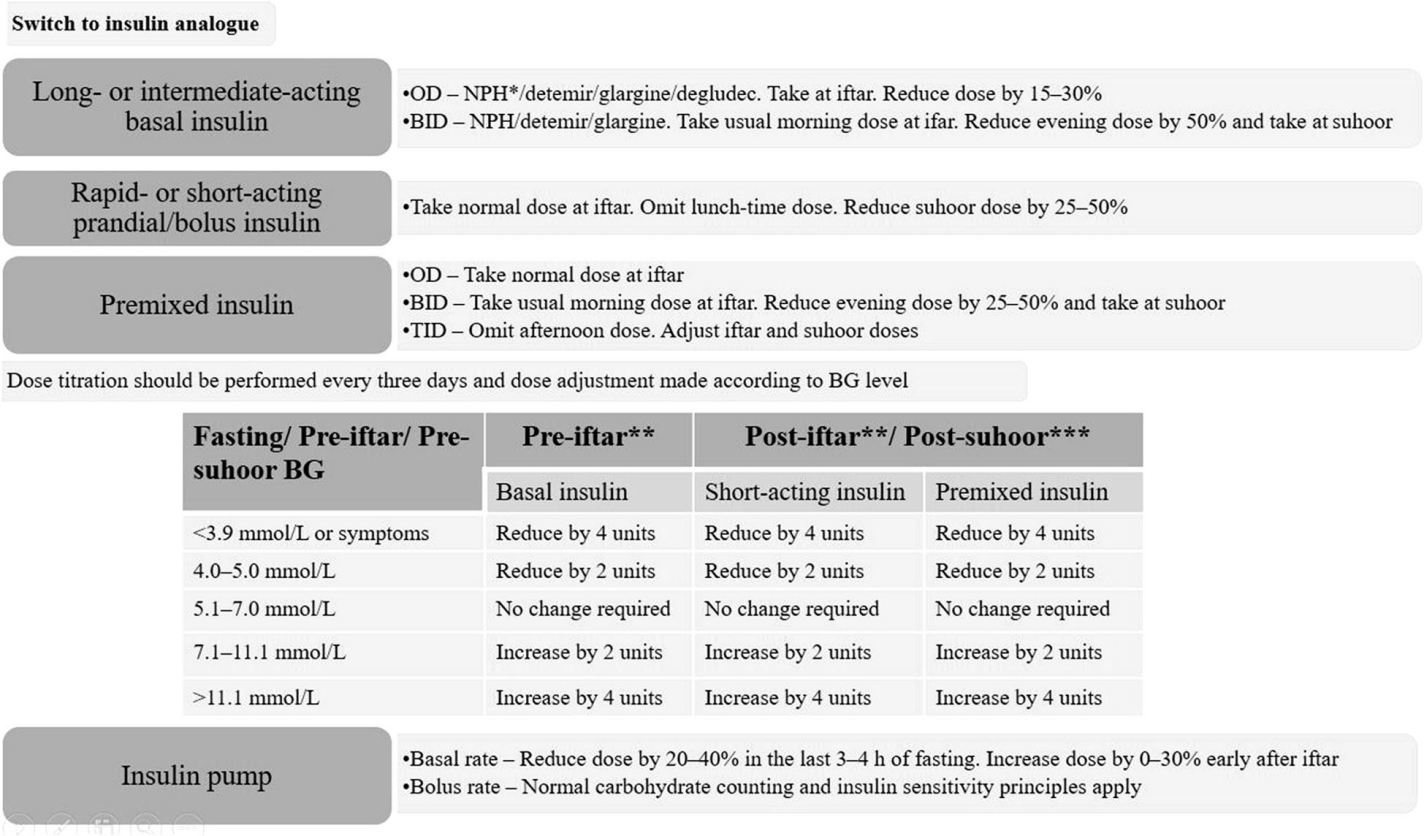
Insulin adjustments and dose titrations in fasting young adults/adolescents with T1DM, and pregnant women during Ramadan. BG blood glucose, BID twice daily, NPH neutral protamine Hagedorn, OD once daily, TID three times a day. *Alternatively, reduced NPH dose can be taken at* suhoor* or at night; **adjust the insulin dose taken before* suhoor*; ***adjust the insulin dose taken before* iftar* Adopted from [122]

Many patients with diabetes, T1DM and T2DM, will fast during religious fasts. The Epidemiology of Diabetes and Ramadan (EPIDIAR) study showed that 42.8% of T1DM subjects and 78.7% of T2DM subjects fasted for at least 15 days during Ramadan [119]. The CREED study reported that 94.2% of T2DM subjects fasted for at least 15 days and 63.6% fasted every day [120]. When fasting, insulin resistance/deficiency can lead to excessive glycogen breakdown and increased gluconeogenesis. This poses a risk of hypoglycemia, hyperglycemia, ketoacidosis, dehydration, and thrombosis [121]. Insulin therapy in religious fasting requires that the patient is educated on the risks posed by fasting, is familiar with SMBG, adheres to appropriate nutrition intake, proper exercise, and dose adjustment to minimize complications [122]. East Africa is lacking in studies to describe the characteristics and multiple approaches to the management of people with diabetes who fast during Ramadan and other religious fasts. Premix insulin analogues have proven efficacy and safety profile with lower rates of hypoglycemia and hence are preferred over premix human insulins in patients with insulin therapy during religious fasting periods [123–126]. Insulin glargine has been safely used in fasting Muslim T2DM patients [127]. Insulin detemir given at 40% of the daily dose at predawn meal (*suhoor*) and 60% as biphasic insulin aspart 30 at sunset (*iftar*) showed non-inferiority when compared with standard care without fasting [128, 129]. Figure 5 gives the recommended insulin adjustments and dose titrations based on SMBG in diabetes, young adults/adolescents with T1DM, and pregnant women during Ramadan. If a patient is taking NPH or premix insulin at *suhoor*, it is important to check blood glucose at noon before up-titration of the pre-*suhoor* dose. If noon blood glucose is < 6.1 mmol/L and pre-*iftar* blood glucose is not at target, a long-acting insulin analogue may be preferred. For those on insulin and SU, a decision on the need to reduce doses of both agents or to start with insulin only is required on the basis of individual assessment. Use of insulin lispro and insulin pumps was reported to be safe in fasting T1DM [130, 131].

#### Religious Fasting Recommendations

- T2DM patients wishing to fast and who are on premix insulin analogue therapy are recommended to use the usual morning dose at sunset (*iftar*) and half the usual evening dose at predawn (*suhoor*) meal, e.g., patients on biphasic insulin aspart 30 with 30 units in morning and 20 units in the evening before Ramadan, the recommended dose will be 30 units in the evening (*iftar*) and 10 units in the morning (*suhoor*) during Ramadan **(Grade A, EL II)**
- In T2DM patients with NPH or premix human insulin at *suhoor*, it is recommended to check blood glucose at noon before up-titration of the *pre*-*suhoor* dose **(Grade B, EL II)**
- Before starting Ramadan or other religious fasts, consider educating the patient with diabetes on risk quantification, blood glucose monitoring, nutrition intake, proper exercise, and dose adjustments to minimize hypoglycemic complications **(Grade A, EL III**)
- Consider advising pregnant women during Ramadan who are on sulfonyl urea therapy and/or insulin to exercise caution because of the high risk of hypoglycemia **(Grade D, EL IV)**

#### Infections

Malaria and other acute febrile illnesses (AFI) are frequent causes of fever in patients with diabetes who reside in East Africa. Hyperglycemia may follow any AFI but equally hypoglycemia has been encountered in malaria and sepsis [132, 133]. In addition, hyperglycemia in patients with AFI may be secondary to medications administered (e.g., steroids). Quinine administration has been associated with hypoglycemia [133]. The glycemic management in patients with AFI should take into consideration compromised GI absorption, renal, hepatic, and cognitive dysfunctions, asthenia, and/or cachexia in such patients [134]. The overall goal is to avoid hypoglycemia [135] and to minimize glycemic variability. Table 7 displays the glycemic management in patients with AFI.

**Table 7:** Glycemic management in patients with acute febrile illness (AFI) Adapted from [134]

	Glycemic management
AFI patients with adequate oral intake	Frequent BGM to check for hyperglycemic episodes Continue OADs in patients eating well if BG is well controlled and no contraindication with OADs Initiate insulin If BG is poorly controlled with OADs
AFI patients with compromised oral intake	Modification in diet (small portion sizes, at frequent intervals)
AFI patients on concomitant corticosteroid therapy	In steroid-induced or worsened hyperglycemia, subcutaneous insulin using a basal or multiple daily injections regimen
AFI patients with compromised hepatorenal function	Rapid-acting insulin in small, frequent doses to manage hyperglycemia
AFI patients with compromised sensorium	Discontinue OADs and initiate IV insulin Alternatively, SC rapid-acting insulin may be used
AFI in elderly patients	Frequent BGM to detect atypical symptoms of hyperglycemia and hypoglycemia
AFI patients with cachexia/asthenia	An insulin regimen which provides both prandial and basal coverage, such as premixed/dual action or basal plus/basal-bolus insulin in patients with lack of energy (asthenia), with or without wasting, loss of weight, muscle atrophy, fatigue, and loss of appetite (cachexia) during the febrile or convalescence phase

*OAD* oral antihyperglycemic drugs, *BGM* blood glucose monitoring, *AFI* acute febrile illness, *IV* intravenous, *IM* intramuscular, *SC* subcutaneous

### Recommendations

- Consider using subcutaneous (SC) insulin with a basal or multiple daily injections regimen in steroid-induced or worsened hyperglycemia **(Grade B, EL III)**
- Consider using Regular insulin or rapid-acting insulin in small, frequent doses to manage hyperglycemia in acute febrile illness patients with metabolic decompensation, compromised hepatorenal function, or cognitive impairment **(Grade B, EL III)**
- Consider using premixed or basal plus/basal-bolus insulin in patients with lack of energy (asthenia), with or without wasting, loss of weight, muscle atrophy, fatigue, and loss of appetite (cachexia) during the febrile or convalescence phase **(Grade B, EL III)**

### HIV Infection and Comorbidities

An increased prevalence of hyperglycemia, insulin resistance, diabetic dyslipidemia, and lipodystrophy has been reported in diabetes patients with HIV infection [136]. Majority of patients presenting with comorbid diabetes and hyperglycemic may be managed as T2DM, taking into consideration comorbidity of infections [137]. Table 8 shows different strategies in the management of diabetes with HIV comorbidity.

**Table 8:** Management of diabetes in patients with HIV Adapted from [137]

Strategies	Management
General management	Treatment for comorbid conditions Hypertension—treatment with ACE inhibitors and ARBs not an optimal choice in patients with HIV Dyslipidemia—treatment with pravastatin, fluvastatin, atorvastatin, and rosuvastatin in patients with HIV
Non-insulin therapies	Use metformin if well tolerated and if no contraindications are present Use SU/alpha-glucosidase inhibitors if metformin is contraindicated/not tolerated. Thiazolidinediones and DPP-4 inhibitors are also used in patients with HIV Use incretin mimetics if weight loss is desired
Insulin	Initiate basal-bolus regimen or premixed insulin (1.0 U/kg/day) at diagnosis Insulin may be tapered or reduced (0.5 U/kg/day) once control is achieved Initiate insulin aspart in patients with ketonuria and for critically ill patients Educate HIV-infected patients on how to dispose of lancets, glucose strips, insulin syringes, pens, and needles to prevent HIV transmission
Changes in HAART	Pre-existing T2DM may continue to be managed after diagnosis of HIV by continuing with the same drug therapy that was being used prior to detection of HIV Patients diagnosed with diabetes and HIV together may be treated with metformin if well tolerated and if no contraindications are present. Depending on the baseline HbA1c, insulin or low dose meglitinides can be initiated as a second-line therapy Patients developing diabetes after HAART may be treated with metformin or other OADs. Insulin is a better and safer choice and may be tapered or reduced once control is achieved

*ACE *angiotensin converting enzyme, *ARB* angiotensin receptor blockers, *HIV* human immunodeficiency virus, *HCW* health care worker, *SU* sulfonylurea, *DPP*-*4* inhibitors dipeptidyl peptidase-4 inhibitors, *HAART* highly active antiretroviral therapy

#### Recommendation

- When insulin therapy is required, consider initiating insulin therapy with basal-bolus regimen or premixed insulin at diagnosis. Higher doses may be needed for control **(Grade C, EL III)**

#### Ketosis-Prone Diabetes

Aggressive diabetes management in patients with ketosis-prone diabetes significantly improves β-cell function and insulin sensitivity often allowing the discontinuation of insulin therapy within a few months of initiation of the treatment. The period of near-normoglycemic remission may last for a few months to several years [138, 139].

### Recommendation

- Initiate insulin as per the clinical situation, but keep a close watch for hypoglycemia. Sudden, significant down-titration of dose frequency and/or requirement may be needed **(Grade C, EL III)**

### Self-Monitoring of Blood Glucose (SMBG)

SMBG in diabetes management helps in making treatment decisions and correcting insulin dose. For optimal use of SMBG, regular review and interpretation by both the patients and physicians is required. In those individuals injecting insulin more than two times per day, SMBG should be done at least three times per day [140]. However, there is insufficient evidence on the number of times SMBG needs to be done in individuals on once-daily insulin therapy, with or without OADs [141]. Evidence from East Africa on the adherence to SMBG has shown suboptimal glycemic control, especially among those who had to pay for glucose strips. For optimal diabetes care in the region, to achieve adherence to SMBG and optimal glycemic control, it has been suggested that patient education be given alongside free glucose strips [142]. Once-daily testing (preferably in the morning) should be done to assess the efficacy of the basal insulin dose. When HbA1c is > 7.0% (> 53 mmol/mol) in spite of a satisfactory FPG, a second test should be performed after the largest meal of the day to exclude postprandial hyperglycemia. In a low-resource setting, it may be considered not cost-effective to perform a postprandial test if the HbA1c is at target [141].

### Recommendation

- Pragmatic suggestions for SMBG should accompany insulin prescriptions **(Grade D, EL IV)**
- Basal insulin is best monitored by FPG, 1–2 times a week **(Grade A, EL II)**
- Prandial insulin is best monitored by paired premeal and postmeal glucose values **(Grade A, EL II)**
- Premixed insulin is initially monitored by prebreakfast and predinner glucose values, followed by postprandial values **(Grade A, EL 2)**

### Exercise

Exercise increases insulin sensitivity, and blood glucose should be checked before and after exercise and appropriate action taken [10].

### Recommendation

- Check blood glucose before and after exercise, and take appropriate action. If exercise is enduring, e.g., bicycle riding, check blood glucose in between exercise and take appropriate action **(Grade A LE I)**

## Hypoglycemia, Weight Gain, and Psychosocial Aspects

### Hypoglycemia

Medications that are associated with the highest risk of injury when used in error are known as high-alert medications. Insulin has long been identified as belonging to this group of medications [143] and patients should be appropriately educated on its use. Hypoglycemia has a significant negative impact on a person’s well-being and QOL and can therefore influence adherence, compliance, and ultimately the success of the insulin therapy. In East Africa, surveys on hypoglycemia indicated high frequency episodes (T1DM, 88%; T2DM, 69%) with almost half of the study population being unaware of the hypoglycemia [144]. Efforts to avoid hypoglycemia include patient education, SMBG, and improving insulin delivery through proper delivery devices and techniques.

### Weight Gain

Insulin therapy is associated with increase in body weight [145]. Patients on insulin therapy should have appropriate physical activity and insulin dose adjustment tailored to carbohydrate intake to avoid excessive weight gain.

### Psychosocial Aspects

Psychosocial barriers to successful insulin therapy in East Africa include lack of physician–patient interaction, understanding of diabetes and its treatments by both physicians and patients, and proper provision of testing and follow-up of patients [146].

#### Educational Motivation/Counseling Support

The patients should be educated on monitoring of glucose, insulin injection technique, insulin storage, recognition/treatment of hypoglycemia, and sick day management by qualified health educators.

#### Mental Illness

Evidence on the association between poor glycemic control and comorbid depression among T2DM patients in East Africa has shown that most T2DM patients with poor glycemic control witnessed further worsening of glycemic control with increasing depression. Insulin-treated T2DM patients with poor glycemic control should be screened for comorbid depression and provided with suitable interventions for optimized diabetes management [147].

## Special Situations

In East Africa, for hospitalized patients with diabetes, hyperglycemia and hypoglycemia are associated with prolonged hospitalization, rehospitalization, increased morbidity, and high mortality [29]. Efforts should therefore be made in hospitalized patients with diabetes to achieve glycemic targets and prevent both hyperglycemia and hypoglycemia.

### Inpatient Settings

Hyperglycemia in hospitalized patients is defined as blood glucose levels of > 7.8 mmol/L [148]. Alterations in diet or changes in antihyperglycemic treatment are warranted when blood glucose levels are persistently above 7.8 mmol/L. If an HbA1c from the previous 3 months is unavailable, measuring the HbA1c in all patients with diabetes or hyperglycemia admitted to the hospital is recommended. On admission, HbA1c value ≥ 6.5% (≥ 48 mmol/mol) implies that diabetes preceded hospitalization, taking into consideration that the patient does not have a hemoglobinopathy, recent episode of malaria, bleeding, or hemolysis [148]. Blood glucose value of 3.9 mmol/L in hospitalized patients indicates hypoglycemia, whereas a blood glucose value of 3.0 mmol/L denotes clinically significant hypoglycemia [149]. Severe hypoglycemia is defined as that associated with severe cognitive impairment regardless of blood glucose level [149].

For the majority of critically ill patients and non-critically ill patients, at a blood glucose value of 10.0 mmol/L insulin therapy should be initiated for treatment of persistent hyperglycemia with the aim of achieving target glucose level in the range between 7.8 and 10.0 mmol/L. More stringent goals (blood glucose values between 6.1 and 7.8 mmol/L) in selected populations in the inpatient setting may occasionally be required and extreme caution taken to avoid hypoglycemia.

Blood glucose monitoring in patients who are eating should be performed before meals, while in patients who are not eating it should be performed every 4–6 h [148]. For patients on IV insulin, blood glucose monitoring should be done every 30 min to 2 h.

#### Treatment for Non-critically Ill Patients

Insulin is the preferred treatment for glycemic control in critically ill patients [148]. For non-critically ill patients with good nutritional intake basal, meal-related, and correction insulin dose are preferred. Subcutaneous rapid- or short-acting insulin before meals or every 4–6 h may be used in patients not on regular meals or in patients receiving continuous enteral/parenteral nutrition to correct hyperglycemia [148]. For non-critically ill patients with poor oral intake or those who are taking nothing by mouth (NPO), basal insulin or a basal plus bolus correction insulin regimen is preferred. Basal-bolus is preferred over sliding scale insulin (SSI) owing to improved glycemic control and reduced hospital complications. Premixed insulin is preferred in the outpatient setting [150] and basal-bolus therapy in the inpatient setting [151].

#### Recommendations

- Consider using basal plus bolus correction insulin regimen, with the addition of meal-related insulin in patients who have good nutritional intake, in non-critically ill patients **(Grade A, EL I)**
- Avoid sliding scale insulin in the inpatient hospital setting **(Grade A, EL I)**

#### Treatment for Critically Ill Patients

In the critically ill patients, continuous IV insulin is preferred. When the patient is able to take regular meals, basal and correction insulin doses are administered. When transitioning T1DM or T2DM patients to outpatient, SC insulin, SC basal insulin should be started 2–4 h before the IV insulin is discontinued.

### Surgery

#### Perioperative Management

**Fig. 6:**
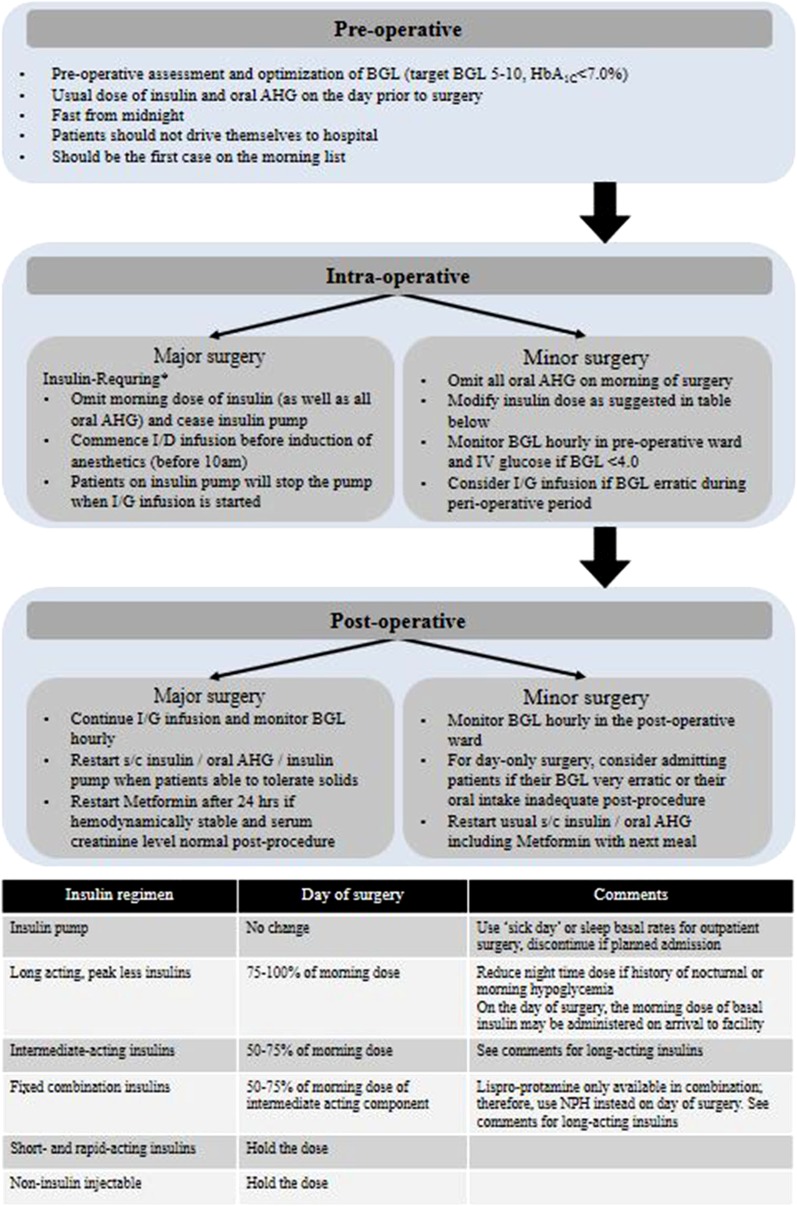
Perioperative management in T1DM and T2DM patients. I/G insulin–glucose infusion, AHG antihyperglycemic agents, BGL blood glucose level. *Includes patients with T1DM as well as insulin-requiring T2DM. Adopted from perioperative diabetes management guidelines, published on the Australian Clinical Practice Guidelines website (https://www.clinicalguidelines.gov.au)

Perioperative management of blood glucose levels is based on the following objectives: (1) reduction in morbidity and mortality, (2) prevention of severe hyperglycemia or hypoglycemia, (3) maintenance of physiological electrolyte and fluid balance, (4) prevention of ketoacidosis, and (5) achieving the target glycemic levels less than 10 mmol/L in critical patients [152, 153] and less than 7.7 mmol/L in stable patients [148]. Long-acting insulin (insulin glargine) should be discontinued 2–3 days prior to surgery and combination of intermediate-acting insulin (NPH) with short- or rapid-acting insulin twice daily or Regular insulin before meals and intermediate-acting insulin at bedtime used for glycemic control. Figure 6 shows the perioperative management in T1DM and T2DM patients.

### Patients on Enteral/Parenteral Nutrition

Three doses of insulin such as basal, meal-related, and correctional insulins are administered in patients on continuous/bolus enteral or parenteral nutrition [154]. In patients on continuous enteral feedings, the basal insulin dose is based on the patient’s preadmission basal insulin dose or 30–50% of the total daily dose (TDD) of insulin or 5 units of NPH/insulin detemir subcutaneously every 12 h or 10 units of insulin glargine every 24 h. The dose of meal-related insulin is calculated as 1 unit of insulin for every 10–15 g carbohydrate per day administered as Regular insulin every 6 h or rapid-acting insulin every 4 h. Correctional insulin should also be administered SC every 6 h using human regular insulin or every 4 h using a rapid-acting insulin such as insulin lispro, insulin aspart, or insulin glulisine. For patients receiving enteral bolus feedings, approximately 1 unit of Regular human insulin or rapid-acting insulin per 10–15 g carbohydrate should be given SC before each feeding. Correctional insulin coverage should be added as needed before each feeding. For patients receiving parenteral nutrition, Regular insulin may be added to the solution, particularly if 20 units of correctional insulin have been required in the past 24 h. A starting dose of 1 unit of human regular insulin for every 10 g carbohydrate is recommended, to be adjusted daily in the solution. Correctional insulin should be administered SC. Table 9 shows insulin dosing for enteral/parenteral nutrition.

**Table 9:** Insulin dosing for enteral/parenteral feedings Adapted from [10]

Situation	Basal/nutritional	Correctional
Continuous enteral feedings	Continue prior basal or, if none, calculate from TDD or consider 5 units NPH/detemir every 12 h or 10 units glargine/degludec daily nutritional: regular insulin every 6 h or rapid-acting insulin every 4 h, starting with 1 unit per 10–15 g of carbohydrate; adjust daily	SC regular insulin every 6 h or rapid-acting insulin every 4 h for hyperglycemia
Bolus enteral feedings	Continue prior basal or, if none, calculate from TDD or consider 5 units NPH/detemir every 12 h or 10 units glargine/degludec daily nutritional: give regular insulin or rapid-acting insulin SQ before each feeding, starting with 1 unit per 10–15 g of carbohydrate; adjust daily	SC regular insulin every 6 h or rapid-acting insulin every 4 h for hyperglycemia
Parenteral feedings	Add regular insulin to TPN IV solution, starting with 1 unit per 10 g of carbohydrate; adjust daily	SC regular insulin every 6 h or rapid-acting insulin every 4 h for hyperglycemia

*IV* intravenous, *SC* subcutaneous, *TDD* total daily dose, *TPN* total parenteral nutrition, *NPH* neutral protamine Hagedorn

### Insulin Therapy in Patients in Their Home Setting

The patients are usually stabilized on the target blood glucose levels before discharge from hospital. Management of insulin therapy while the patient is at home includes education about insulin regimens, appropriate choice of regimen, scheduling regular blood glucose monitoring, awareness on hypoglycemic symptoms and their management, contact details of the health care worker in the nearest health care facility in case of emergency, and a regular follow-up within 10–14 days’ time.

## Practical Aspects

### Insulin Delivery Devices

Insulin can be administered via various methods such as vial and syringe, insulin pen, jet injectors, and continuous subcutaneous insulin infusion (CSII) using insulin pumps. Only inhaled insulin (Afrezza) is not injectable [155]. In East Africa, vial and syringe, insulin pens, and insulin pumps are accessible. The choice of insulin delivery device should be individualized for patients.

### Recommendation

- Health care authorities and planners should be alerted to the risks associated with syringe or pen needles 6 mm or longer in children **(Grade A, LE II)**

### Insulin Transport and Storage

Specific storage conditions provided by the manufacturer in the package inserts (stored for 28 days at 30 °C; 45 days at 25 °C) should be followed. Insulin should be stored in a cool (< 30 °C) environment and must be protected from extremes of temperature such as direct sunlight, kitchen, glove box of a car, over the engine in a motor vehicle, or left in a closed stationary motor vehicle.

Insulin can be safely transported from the health facility or pharmacy in a bag that will not be exposed to excessive high temperatures. If exposure to high temperatures is envisaged, it is advised to transport the insulin in the original packaging placed on an icepack.

### Recommendation

- Transportation of insulin from a health facility to home can be safely done without an icepack if no extremes of temperatures are envisaged. If uncertainty exists about an exposure to high temperatures (> 30 °C) it is advised to transport insulin on an ice pack **(Grade A, LE IV)**

**Fig. 7:**
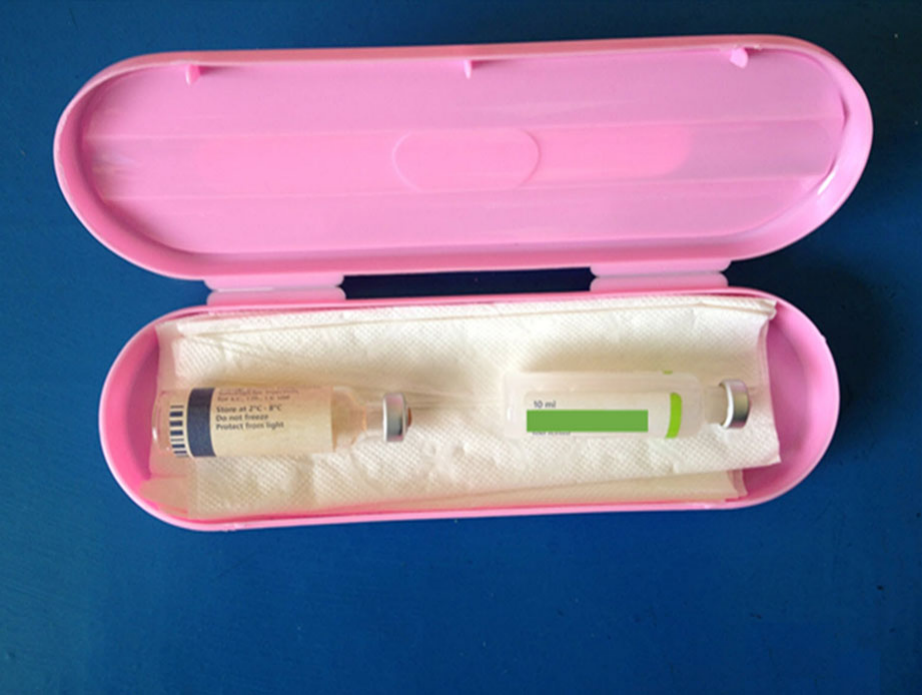
Clean boxes used to safely keep insulin and insulin syringes at home Image courtesy of Silver Bahendeka

Lack of refrigeration facilities at home may force patients to use improvised *cooling systems* such as storing insulin vials submerged in water or keeping insulin in a pot with sand [156]. These should be used with caution as they may result in contamination of insulin and subsequent injection abscesses. Figure 2 shows *insulin tattoos* following the injection of contaminated insulin stored in a water container. The patient also overslanted the needle. When a reliable refrigerator is not available, it is advisable to follow the manufacture’s advice in the package inserts and store insulin in a clean container at room temperature, in a clean environment (a cupboard and not under a bed), until more studies are available on insulin storage. Figure 7 shows such a container where insulin can be safely kept in a refrigerator or at room temperature in a cupboard.

### Injection Sites

Correct technique in insulin delivery is critical for optimal control of diabetes. Various factors are considered while injecting insulin, including choice of site, injection site rotation, skin fold, and injection techniques. The recommended injection and infusion sites are the abdomen, thigh, buttock, and upper arm [157].

### Recommendation

- Recommended injection and infusion sites are the abdomen, thigh, buttock, and upper arm **(Grade A, LE I)**

### Needle Length

A 4-mm needle is long enough to traverse the skin and enter the SC tissue, with little risk of IM or intradermal injection. Therefore, it is considered the safest needle for adults and children regardless of age, sex, ethnicity, or body mass index (BMI). A 5-mm needle may be acceptable in obese individuals. Very young children (≤ 6 years old) and very thin adults should use the 4-mm needle by lifting a skin fold and inserting the needle perpendicularly into it. Others may inject using the 4-mm needle without lifting a skinfold. Previously needle lengths that were recommended for SC injections were ≥ 8 mm for adults and ≥ 6 mm for children. These are now considered to be too long because they increase the risk of IM injections without evidence of improved control [157]. The 4-mm needle should be inserted perpendicular to the skin, at 90° to the skin surface, not at an angle, regardless of whether a skinfold is raised. Currently only the 6-mm and larger syringe needles are available in East Africa. Raising the skin fold is therefore recommended until the 4-mm needles are available.

#### Recommendation

- A 4-mm needle is recommended for all patients with diabetes **(Grade A, LE I)**
- The safest currently available syringe needle for all patients is 6 mm in length. However, when any syringe needle is used in children ≥ 6 years old, adolescents, or slim to normal weight adults (body mass index of 19–25 kg/m^2^—calculated as the weight in kilograms divided by the height in meters squared) injections should always be given into a lifted skin fold at 90° **(Grade A, LE I)**

#### Injection Site Complications

Injection site complications include injection site infection and abscesses, injection site skin scarification (*insulin tattoos*), and lipohypertrophy. Local skin injection site complications are common in patients on insulin therapy in East Africa, because of poor insulin storage (keeping insulin in water to keep it cool), and wrong injection technique (overslanting the needle resulting in insulin being injected intradermally). Lipohypertrophy is a thick soft to firm swelling with rubbery consistency, which appears on the surface of the skin at the site of insulin injection. Injection sites should be rotated.

#### Recommendation

- Injections should be systematically rotated to avoid lipohypertrophy. This means at least 1 cm (1-finger breadth) from previous injections **(Grade A, LE II)**
- Injection sites should be examined by the health care worker at least once a year and preferably every visit **(Grade A, LE II)**
- After the insulin has been pushed in, patients should count slowly to 10 and then withdraw the needle from the skin. This is necessary to prevent medication leakage so as to get the full dose **(Grade A, LE I)**
- Many patients in East Africa reuse syringes for various reasons, including financial. This is not recommended by the manufacturer. There is an association between needle reuse and lipohypertrophy. Patients who reuse needles should not be subjected to alarming claims of excessive morbidity from this practice **(Grade A, LE III)**

#### Needle Stick Injuries

Needle stick injuries are common among physicians and health care workers and warrant training on preventive methods. Health care workers in Africa suffer 2–4 needle-stick injuries per year on average, with Nigeria and Tanzania reporting 2.10 injuries per health care worker on average [158], and this should be avoided.

#### Recommendation

- Safety injection devices should be considered first-line choice if injections are given by a third party. Pens and syringes with needles used in this setting should have protective mechanisms for all sharp ends of the delivery device **(Grade A, LE II)**
- The health care worker should be involved in the training of the patient and safe disposal of sharps **(Grade A, LE I)**

#### Lipoatrophy

Lipoatrophy is clinically characterized by visible cutaneous depression and palpable atrophy of SC fat tissue at the injection site. It is an immunological response to insulin aggregates in the presence of high circulating titers of anti-insulin autoantibodies. Rotation of injection sites minimizes this complication.

#### Pain

Pain is the commonest adverse event associated with insulin use. This is especially so with wrong injection technique and reuse of needles and syringes.

### Barriers and Myths Concerning Insulin

Educating and training patients on diabetes self-management can address patient-related barriers to insulin use. Barriers affecting physicians and health care workers could be addressed through programs such as skill enhancement and conferences. In addition, drug or device-specific barriers can be addressed through continuing education programs, flexible insulin regimens, preparations, and modern devices.

## Biosimilar Insulins

For many decades, the major sources for (animal) insulin were pancreata of pigs and cows. After discovery of the primary structure of the insulin molecule, insulin was manufactured by recombinant technology. This paved the way for pharmaceutical companies other than the *original* multinational ones to also manufacture and market similar insulins. Differences in the manufacturing processes (none of the insulin manufacturing procedures are identical) result in the fact all insulins that might become biosimilar differ from the originator insulin to some extent [159]. The current unanswered question is: do such differences in the structure of the insulin molecule and/or the purity and so on have clinically relevant consequences? The European Union guidelines for market approval require that the manufacturer demonstrate that the insulin has a safety and efficacy profile that is similar to that of the *original* insulin formulation. Biosimilar insulins are available on the East African market. Transferring from one insulin another requires a dose titration. There is no published data on the consequences of switching from one insulin to another in patients on insulin therapy in East Africa. However, observations from diabetes clinics indicate a need for titrating dosages at every switch.

### Recommendation

- In switching from one insulin to another similar insulin (*original to biosimilar*) it is advised to carry out a dose titration always starting with a reduced dose and to up-titrate to avoid hypoglycemia. Self-monitoring of blood glucose is therefore necessary **(Grade D, LE V)**

## Benefits of Early Optimized Control of Diabetes

### Economic Consideration

The high negative impact of diabetes on the economy of individuals, families, societies, and countries has been well established [2]. The direct costs relate to the cost of treatment of disease and related complications, while the indirect costs are associated with income losses through reduced productivity and disability. The present data on per patient expenditures for diabetes in SSA are extrapolated from the western world data. Accordingly, direct cost estimated per patient expenditure for diabetes in 2015 in SSA ranged between US$243 and US$419 [1]. A recent study data from LMIC showed average per patient costs of US$580 [160]. The percentage of indirect costs for the East African region was at 39.7% [161]. Diabetes is associated with catastrophic family expenditure; patients, family, and other care takers have to pay out-of-pocket to meet the expenses related to diabetes management [30]. While public health facilities in East Africa offer insulin for free, the supply is usually insufficient or the distance travelled to access the free insulin is more costly than the cost of insulin in the private sector. Insulin syringes are usually supplied in quantities that would be insufficient for single use. Glucose meters for SMBG are not available from the public health sector. These areas are of public concern and need to be urgently addressed by the health authorities as they adversely affect the outcome of diabetes management.

### Hit Early Hit Hard Paradigm Shift

The conventional stepwise therapy approach in T2DM involves contributing factors like abnormal gastric emptying, insulin resistance, dysfunctional lipid metabolism, excess hepatic glucose production, β-cell failure, poor α-cell regulation, and altered role of the kidney in handling glucose being addressed one-by-one in a sequential fashion. Current data suggests that the stepwise therapy approach to achieve glycemia, blood pressure, and lipid targets, despite significant improvements in the glycemic control and lipids, is associated with disappointingly low percentages of subjects attaining all three targets. This irregular pattern in glycemic control is due to lower control rates for all three targets [162]. Furthermore, this approach may lead to a decline in β-cell function.

Moreover, clinical inertia in the stepwise approach therapy may expose T2DM patients to prolonged periods of hyperglycemia of even up to 8 years [163]. Intervals between adding or switching agents were previously much longer than currently recommended by the ADA/EASD and AACE [17, 104]. There still exists a difference of opinion between ADA/EASD and AACE guidelines on the management of T2DM in patients with HbA1c level of 7.5–9.0% (58–75 mmol/mol) at diagnosis with regard to insulin therapy versus the addition sulfonyl urea. The 6-year-long Outcome Reduction with Initial Glargine Intervention (ORIGIN) study showed that at diagnosis of T2DM, early initiation of insulin in combination with OADs resulted in a stable pattern of glycemic control [164]. Several other studies have reported the beneficial effects on β-cell functions in patients with T2DM who were on early and aggressive treatment with insulin in combination with OADs [165–167].

Currently there are no studies that show the comparative efficacy of early initiation of insulin in combination with OADs versus stepwise addition of therapy over time. However, longer-term follow-up observational study in UKPDS [83] showed that better outcomes are possible by initiating intensive glycemic control in early stages of diabetes, preferably from the start of the diagnosis of diabetes. While there is still a need for more long-term studies, it appears that *hit early hit hard* with use of multiple therapies, including insulin, may alter the natural history of β-cell function in patients with T2DM.

## Research Agenda

The World Health Organization’s STEPwise approach to surveillance (STEPS) data showed that there is a high rate of undetected diabetes in the East African region [23–28]. The high rates of undetected diabetes imply a likelihood of late presentation with complications in a health system already overburdened by high prevalence of infectious diseases, including HIV/AIDS, TB, and malaria. Overall, there is increased risk of adverse outcomes in diabetes patients in the East African region. Moreover, there are significant challenges in accessing diagnosis and treatment for diabetes, complicated by social, cultural, and ethnic factors.

This guideline recommends safely achieving tight glucose control by lifestyle modifications and achieving target values for HbA1c, FPG, PPG, hypoglycemia, nocturnal hypoglycemia, and glycemic variability in patients with diabetes, for optimized diabetes management. The guideline recommends intensive diabetes therapy in T1DM with basal-bolus (basal prandial) regimens that mimic the normal β-cell insulin secretion. For the treatment management in T2DM, the guideline recommends individualized glycemic goals based on FPG, PPG, and HbA1c levels. In addition, the guideline recommends initiation with once-daily basal insulin, once-daily premixed/co-formulation insulin, or twice-daily premixed insulin, either alone or in combination with GLP-1 RA (where available) or in combination with other OADs to achieve the glycemic goals and prevent long-term complications. The guideline has identified key concepts in optimal glycemic control in T2DM that included choice of an appropriate insulin regimen and stepwise approach of insulin initiation, titration, and intensification. The strength of the guideline is that the recommendations put forth are based on the existing established guidelines and published evidence. East Africa lacks sufficient evidence from RCTs and even data from small studies to support the use of insulin in special populations such as diabetes in pregnancy; diabetes and lactation; diabetes and renal, cardiac, and hepatic impairment; monogenic diabetes; and management in special situations like Ramadan and other faith-based fasting and acute and chronic infections. Therefore, the EADSG Guidelines Development Task Force resorted to an evidence-based consensus to arrive at the guidelines for use of insulin in such special populations and special situations.

The lack of reliable data on insulin use in the region necessitates the need for high-quality studies to be carried out in East Africa; they will help in making treatment decisions in diabetes management.

We hope that the current guidelines will be a useful reference tool to all health care professionals in East Africa and will lead to an improvement in the optimal care of diabetes in the region. These guidelines will be updated with respect to newer evidence and newer insulin formulations that will be available on the East African market in the near future and based on further observational research, involving large numbers of physicians and in the setting of routine outpatient care of diabetes in the Eastern Africa region.
